# The minimizer Jaccard estimator is biased and inconsistent

**DOI:** 10.1093/bioinformatics/btac244

**Published:** 2022-06-27

**Authors:** Mahdi Belbasi, Antonio Blanca, Robert S Harris, David Koslicki, Paul Medvedev

**Affiliations:** Department of Computer Science and Engineering, The Pennsylvania State University, University Park, PA, USA; Department of Computer Science and Engineering, The Pennsylvania State University, University Park, PA, USA; Department of Biology, The Pennsylvania State University, University Park, PA, USA; Department of Computer Science and Engineering, The Pennsylvania State University, University Park, PA, USA; Department of Biology, The Pennsylvania State University, University Park, PA, USA; Huck Institutes of the Life Sciences, The Pennsylvania State University, University Park, PA, USA; Department of Computer Science and Engineering, The Pennsylvania State University, University Park, PA, USA; Huck Institutes of the Life Sciences, The Pennsylvania State University, University Park, PA, USA; Department of Biochemistry and Molecular Biology, The Pennsylvania State University, University Park, PA, USA

## Abstract

**Motivation:**

Sketching is now widely used in bioinformatics to reduce data size and increase data processing speed. Sketching approaches entice with improved scalability but also carry the danger of decreased accuracy and added bias. In this article, we investigate the minimizer sketch and its use to estimate the Jaccard similarity between two sequences.

**Results:**

We show that the minimizer Jaccard estimator is *biased* and *inconsistent*, which means that the expected difference (i.e. the bias) between the estimator and the true value is not zero, even in the limit as the lengths of the sequences grow. We derive an analytical formula for the bias as a function of how the shared *k*-mers are laid out along the sequences. We show both theoretically and empirically that there are families of sequences where the bias can be substantial (e.g. the true Jaccard can be more than double the estimate). Finally, we demonstrate that this bias affects the accuracy of the widely used mashmap read mapping tool.

**Availability and implementation:**

Scripts to reproduce our experiments are available at https://github.com/medvedevgroup/minimizer-jaccard-estimator/tree/main/reproduce.

**Supplementary information:**

Supplementary data are available at *Bioinformatics* online.

## 1 Introduction

Sketching is a powerful technique to drastically reduce data size and increase data processing speed. Sketching techniques create a smaller representation of the full dataset, called a *sketch*, in a way that makes algorithms more efficient, ideally without much loss of accuracy. This property has led to sketching methods being increasingly used to meet the scalability challenges of modern bioinformatics datasets, though sometimes without understanding the detrimental effects on accuracy.

A thorough treatment of sketching in bioinformatics can be found in the excellent surveys of Rowe (2019) and Marçais *et al.* (2019a), but we mention a few notable examples next. The seminal Mash paper (Ondov *et al.*, 2016) showed how estimating the Jaccard similarity of two sequences from their minhash sketches (Broder, 1997) enables clustering of sequence databases at unprecedented scale. The hyperloglog sketch (Flajolet *et al.*, 2007) is used to compute genomic distances (Baker and Langmead, 2019); the modulo sketch (Schleimer *et al.*, 2003) is used to search sequence databases (Pierce *et al.*, 2019); strobemers (Sahlin, 2021) and minhash with optimal densification (Shrivastava, 2017; Zhao, 2019) are used for sequence comparison; order minhash is used to estimate edit distance (Marçais *et al.*, 2019b); and count minsketch (Cormode and Muthukrishnan, 2004) is used for *k*-mer counting (Crusoe *et al.*, 2015).

One of the most widely used sketches, which forms the basis of our work, is the minimizer sketch (Roberts *et al.*, 2004; Schleimer *et al.*, 2003), which selects, for each window of *w* consecutive *k*-mers, the *k*-mer with the smallest hash value. Minimizer sketches are used for transcriptome clustering (Sahlin and Medvedev, 2020) and error correction (Sahlin and Medvedev, 2021), as well as for seed generation by the Peregrine genome assembler (Chin and Khalak, 2019) and the widely used minimap (Li, 2016, 2018) and mashmap (Jain *et al.*, 2017, 2018a) aligners.

Just as with other sketching techniques, in order for the minimizer sketch to be useful, it must come with theoretical (or at least empirical) bounds on the loss of accuracy that results from its use. For instance, the minhash Jaccard estimator used by Mash has the property of being *unbiased* (Broder, 1997), i.e. its expected value is equal to the true Jaccard. Such a theoretical guarantee, however, cannot be assumed for other sketches. Here, we will consider the example of the *minimizer Jaccard estimate* (Jain *et al.*, 2017, 2018a,b), which computes the Jaccard similarity using minimizer sketches and forms the basis of the widely used mashmap (Jain *et al.*, 2017, 2018a) aligner. This estimator is useful for sequence alignment because the minimizer sketch has the nice property that, roughly speaking, the sketch of a long string contains the sketches of all its substrings. However, its theoretical accuracy has not been studied and empirical evaluations have been limited.

In this article, we study the accuracy of the minimizer Jaccard estimator $\hat{J}$, both theoretically and empirically. We prove that $\hat{J}$ is in fact biased and inconsistent (i.e. the bias is not zero, and it remains so even as the sequences lengths grow). We derive an approximate formula for the bias that is accurate up to a vanishingly small additive error term, and give families of sequence pairs for which $\hat{J}$ is expected to be only between 40% and 63% of the true Jaccard. We then empirically evaluate the extent of the bias and find that in some cases, when the true Jaccard similarity is 0.90, the estimator is only 0.44. We also study both theoretically and empirically the bias of $\hat{J}$ for pairs of sequences generated by a simple mutation process and find that, while not as drastic, the bias remains substantial. Finally, we show that the bias affects the mashmap aligner by causing it to output incorrect sequence divergence estimates, with up to a 14% error. Our results serve as a cautionary tale on the necessity of understanding the theoretical and empirical properties of sketching techniques.

## 2 The minimizer sketch and minimizer Jaccard estimator

In this section, we will define the minimizer sketch (Roberts *et al.*, 2004; Schleimer *et al.*, 2003) and the Jaccard estimator derived from it (Jain *et al.*, 2017). Let *k *>* *2 and *w *>* *2 be two integers. This article will assume that we are given two duplicate-free sequences *A* and *B* of *L k*-mers, with L≥7(w+1). A sequence is *duplicate-free* if it has no duplicate *k*-mers, but *A* and *B* are allowed to share *k*-mers. These requirements on the sequences do not limit the general scope of our results. In particular, since we will show the existence of bias for these constrained cases, it immediately implies the existence of bias within broader families of sequences.

Let *A_i_* denote the *k*-mer starting at position *i* of *A*, with *A*_0_ and $A_{L-1}$ being the first and last *k*-mers, respectively. Let ${\text{Sp}}^{k}(A)$ be the set of all *k*-mers in *A*. We define *I*(*A*, *B*) to be the number of *k*-mers shared between *A* and *B*, and *U*(*A*, *B*) to be the number of *k*-mers appearing in either *A* or *B*. Formally,

$$\begin{array}{c} I(A,B)≜|{\text{Sp}}^{k}(A)\cap {\text{Sp}}^{k}(B)|\\ U(A,B)≜|{\text{Sp}}^{k}(A)\cup {\text{Sp}}^{k}(B)| \end{array}$$

The Jaccard similarity between the sequences *A* and *B* is defined as

$$J(A,B)≜\frac{I(A,B)}{U(A,B)}.$$

Suppose we have a hash function *h* that takes an element from the set of all *k*-mers and maps it to a real number drawn uniformly at random from the unit interval [0,1]. Under this hash function, the probability of a collision is 0. We denote by *a_i_* the hash value assigned to *k*-mer *A_i_* and for integer w≥2 define the minimizer sketch of *A* as

$$\text{MS}(A;w)≜\cup_{i=0}^{L-w}\{A_{p}:p=\text{arg}\;\underset{j\in [i,i+w-1]}{\text{min}}a_{j}\}.$$

An element in MS(A;w) is called a *minimizer* of *A*. The minimizer intersection and the minimizer union of *A* and *B* are defined, respectively, as

$$\begin{array}{c} \hat{I}(A,B;w)≜|\text{MS}(A;w)\cap \text{MS}(B;w)|\\ \hat{U}(A,B;w)≜|\text{MS}(A;w)\cup \text{MS}(B;w)|. \end{array}$$

The minimizer Jaccard estimator between *A* and *B* is defined as

$$\begin{array}{c} \hat{J}(A,B;w)≜J(\text{MS}(A;w),\text{MS}(B;w))\\ =\frac{\hat{I}(A,B;w)}{\hat{U}(A,B;w)}. \end{array}$$

## 3 Main theoretical results

In this section, we state our main theoretical results and give some intuition behind them. We can think of the relationship between the shared *k*-mers of *A* and *B* as the subset of $\left(A_{0},\ldots ,A_{L-1})\times (B_{0},\ldots ,B_{L-1}\right)$ that corresponds to pairs of equal elements; i.e. to pairs (*A_i_*, *B_j_*) with *A_i_* = *B_j_*. Because *A* and *B* are duplicate-free, this relationship is a matching. We call this the *k-mer-matching* between *A* and *B*. Our main result is stated in terms of a term denoted by B(A,B;w), which is a deterministic function of the window size *w* and of the *k*-mer-matching between *A* and *B*. We postpone the exact definition of B(A,B;w) until Supplementary Appendix A.1, since it requires the introduction of cumbersome notation. The main technical result of this article is:

Theorem 1. *Let* w≥2, k≥2*, and* L≥7(w+1)  *be integers. Let A and B be two duplicate-free sequences, each consisting of L k-mers. Then there exists* $\varepsilon \in [0,\frac{15w^{2}}{\sqrt[{3\;}]{L}}]$  *such that*
 

$$B(A,B,w)-\varepsilon \leq E[\hat{J}(A,B;w)]-J(A,B)\leq B(A,B,w)+\varepsilon .$$

In other words, the difference between the expected value of the minimizer Jaccard estimator and the true Jaccard is B(A,B;w), up to a vanishingly small additive error. We now investigate the value of the term B, which approximates the bias. First, we can show that for padded sequences, B(A,B;w)<0, except that B(A,B;w)=0 when J(A,B)=0. We say two sequences are padded if they do not share any minimizers in the first or last *w k*-mers. (We note that the effect of padding becomes negligible for longer sequences.)

Theorem 2. *Let* w≥2, k≥2*, and* L≥7(w+1)  *be integers. Let A and B be two duplicate-free padded sequences, each consisting of L k-mers. Then* B(A,B;w)<0  *unless* J(A,B)=0*; when* J(A,B)=0*, we have* B(A,B;w)=0.

Moving forward, we may omit *A*, *B*, and *w* from our notation when they are obvious from the context. Theorems 1 and 2 state that $\hat{J}$ is biased for padded sequences as long as *ε* is sufficiently small (e.g. *L* is sufficiently large or *w* is sufficiently small). Here, we use ‘biased’ in the statistical sense that $E[\hat{J}]\neq J$. Intuitively, $\hat{J}$ is biased because it depends on the layout of the shared *k*-mers along the sequences (i.e. on the *k*-mer-matching), while *J* only depends on the number of shared *k*-mers but not on their layout. Note that our results hold for any duplicate-free choice of *A* and *B* and do not assume any background distribution, e.g. that *A* is generated uniformly at random.

**Fig. 1:**
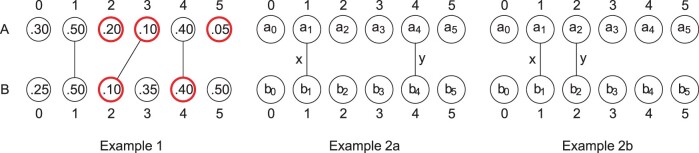
Examples of the Jaccard and the minimizer Jaccard estimator. Each example shows the *k*-mers of a sequence *A* on top, the *k*-mers of a sequence *B* on the bottom and lines connecting *k*-mers show the *k-mer-matching* between *A* and *B*. Each *k*-mer is labeled by its hash value. In Example 1, J(A,B)=1/3. The minimizers for *w *=* *3 are circled in bold red. Here, $\hat{I}(A,B;3)=1,\;\hat{U}(A,B;3)=4$, and $\hat{J}(A,B;3)=1/4$. Examples 2a and 2b give intuition for why the minimizer Jaccard estimator is biased. Here, *a_i_* refers to the hash value assigned to position *i* and *x* and *y* are *k*-mers shared between *A* and *B*. The expected minimizer Jaccard for *w *=* *2 is different in the two examples but the Jaccard is not (*J *=* *0.2); hence the expected minimizer Jaccard cannot be equal to the true Jaccard. (A color version of this figure appears in the online version of this article.)

We illustrate the point with Examples 2a and 2b in Figure 1. In both examples, the expected size of $\hat{I}$ is the probability that *x* is a minimizer in *A* and in *B* plus the probability that *y* is a minimizer in *A* and in *B*. These two probabilities are equal to each other in these examples and $E[\hat{I}]=2\tau$, for some *τ*. When *w *=* *2, in Example 2a, *τ* is the probability that *a*_1_ is (i) not larger than both *a*_0_ and *a*_2_, and (ii) not larger than *b*_0_ and *b*_2_. Since this statement is about the ordering of five independently chosen hash values, a straight-forward enumeration gives that τ=64/120. In Example 2b, however, *b*_2_ = *a*_2_, and *τ* is the probability that *a*_1_ is (i) not larger than both *a*_0_ and *a*_2_ and (ii) not larger than both *b*_0_ and *a*_2_. This statement is now about the order of four (not five) independently chosen hash values, and an enumeration gives τ=14/24. Hence, the values of *τ* are different in the two examples, and therefore $E[\hat{I}]$ is also different.

The discrepancy on $E[\hat{I}]$ turns out to be crucial since it induces a bias. Specifically, as part of the proof of Theorem 1, we will show that $E[\hat{J}]\approx \frac{E[\hat{I}]}{\frac{4L}{w+1}-E[\hat{I}]}$, and, since the difference between the expected sizes of the minimizer intersections varies for the two examples, we have that $E[\hat{J}]$ is also different; in particular, $E[\hat{J}]$ is affected by the layout of the *k*-mer-matching. Note, however, that the Jaccard similarity in both examples is the same, with *J *=* *0.2, leading to the intuition that $\hat{J}$ is biased when *w *=* *2. Theorems 1 and 2 show that this bias extends beyond this contrived example and holds for most sequences of interest.

Next, we consider the value of B(A,B;w) for some more concrete families of sequence pairs. First, consider the case where any pair of *k*-mers that are shared between *A* and *B* are separated by at least *w* positions. This may approximately happen in practice when *A* and *B* are biologically unrelated and the *k*-mer matches are spurious. Formally, we say two padded sequences *A* and *B* are *sparsely-matched* if for all *p* and *q* such that *A_p_* = *B_q_*, $\left\{A_{p-w},\ldots ,A_{p-1},A_{p+1},\ldots ,A_{p+w}\}\notin {\text{Sp}}^{k}(B\right)$, and $\left\{B_{q-w},\ldots ,B_{q-1},B_{q+1},\ldots ,B_{q+w}\}\notin {\text{Sp}}^{k}(A\right)$. In such a case, one could imagine that since the shared *k*-mers do not interfere with each other’s windows, the estimator might be unbiased. It turns out this is not the case.

Theorem 3. *Let* w≥2, k≥2*, and* L≥7(w+1)  *be integers. Let A and B be two duplicate-free, padded, sparsely-matched sequences, each consisting of L k-mers. Then* $B(A,B;w)\leq -J(A,B)\frac{3w^{2}-3w}{8w^{2}-2}$.

A direct consequence of combining this with Theorem 1 is that for sparsely-matched sequences with J(A,B)>0,

$$\frac{E[\hat{J}(A,B;w)]}{J(A,B)}\leq \frac{5w^{2}-3w-2}{8w^{2}-2}+\frac{\varepsilon }{J(A,B)}.$$

For example, for *w *=* *20 and sufficiently long sequence pairs with a fixed (i.e. independent of *L* or *w*) Jaccard similarity, $\hat{J}$ is at most 61% of the true Jaccard. The bias cannot be fixed by changing *w*, since at *w *=* *2, $\hat{J}$ is at most 40% of *J*, and, as *w* grows, $\hat{J}$ is at most 63% of the true Jaccard. This example also shows that $\hat{J}$ is not only biased but also *inconsistent*, i.e. $E[\hat{J}]$ does not converge to *J* even as the sequences grow long.

Let us now consider the opposite side of the spectrum, where instead of being sparsely-matched, *A* and *B* are related by the simple mutation model (i.e. every position is mutated with some constant probability (Blanca *et al.*, 2021)). Deriving the bias for this case proved challenging, since the mutation process adds another layer of randomness. Instead, we derive the bias in a simpler deterministic version of this process, where there is a mutation every *g* positions, for some g>w+2k.

Theorem 4. *Let* 2≤w<k, g>w+2k*, and* L=ℓg+k  *for some integer* ℓ≥1*. Let A and B be two duplicate-free sequences with L k-mers such that A and B are identical except that the nucleotides at positions* k−1+ig*, for* i=0,…,ℓ*, are mutated. Then*,

$$B(A,B;w)=\frac{2\ell (\ell g+k)h(w)}{\left(\ell (g+k)+2k-\ell h(w))(\ell (g+k)+2k\right)},$$

*where* $h(w)=\frac{\left(w+1)(1-2(H_{2w}-H_{w})\right)}{2}$  *and* $H_{n}=\sum_{j=1}^{n}\frac{1}{j}$  *denotes the n-th Harmonic number.*

We can use this theorem in combination with Theorem 1 to obtain a precise approximation of the bias of $\hat{J}$ for this family of sequences. For instance, taking *k *=* *15, *w *=* *10, *L *=* *9992, and *g *=* *43 yields that $\hat{J}$ is ≈10% smaller than the true Jaccard. As *g* increases, the bias decreases, e.g. for *g *=* *100 and *L *=* *10, 016, $\hat{J}$ is 4% smaller than the true Jaccard.

## 4 Overview of Theorem 1 proof

Due to space constraints, we will focus only on the main theorem (Theorem 1) in the main text, providing the intuition and giving an overview of the technical highlights. The proofs of all the theorems, as well as all the building blocks, are deferred to the Supplementary Appendix. Our main technical novelty is the derivation of a mathematical expression, C(A,B;w), that approximates the expected value of the size of the minimizer intersection $\hat{I}(A,B;w)$ between two sequences *A* and *B*.

Lemma 1. $C(A,B;w)\leq E[\hat{I}(A,B;w)]\leq C(A,B;w)+2$.

C(A,B;w) is function of *w*, *L*, and of the *k*-mer-matching between *A* and *B*. In particular, when these parameters are known, then C(A,B;w) can be easily computed. We define C(A,B;w) formally in Supplementary Appendix A.1, since it requires the introduction of additional notation. In Supplementary Appendix 4.1, we give a high level proof of overview of Lemma 1 that does not require the definition of C.

To prove Theorem 1, we first use Lemma 1 to approximate the value of $E[\hat{J}(A,B;w)]$.

Lemma 2. *Let* w≥2, k≥2*, and* L≥7(w+1)  *be integers. Let A and B be two duplicate-free sequences, each consisting of L k-mers. Then there exists* $\varepsilon \in [0,\frac{15w^{2}}{\sqrt[{3\;}]{L}}]$  *such that*
 

$$\frac{C(A,B;w)}{dL-C(A,B;w)}-\varepsilon \leq E[\hat{J}(A,B;w)]\leq \frac{C(A,B,w)}{dL-C(A,B;w)}+\varepsilon ,$$

where d=4/w+1.

Section 4.2 provides a sketch of the proof. Finally, to prove Theorem 1, we show that

$$B(A,B,w)\approx \frac{C(A,B,w)}{dL-C(A,B;w)}-J(A,B),$$

up an additive error that vanishes as the number of *k*-mers growths; when combined with Lemma 2 this approximation yields Theorem 1 immediately. In the following subsection, we will use $\hat{I}$ as shorthand for $\hat{I}(A,B;w)$; we will similarly use $\hat{U},\hat{J},C$.

### 4.1 Lemma 1

In this section, we give an intuition for the proof of Lemma 1 and for where C(A,B;w) comes from. Let $M_{p}^{A}$ be the indicator random variable for the event that *A_p_* is a minimizer in *A*. The expected size of the minimizer intersection can then be written in terms of $M_{p}^{A}$ as follows:

(1) $$\hat{I}(A,B;w)=\sum_{p=0}^{L-1}\sum_{q=0}^{L-1}M_{p}^{A}M_{q}^{B}1(A_{p}=B_{q})$$

**Fig. 2:**
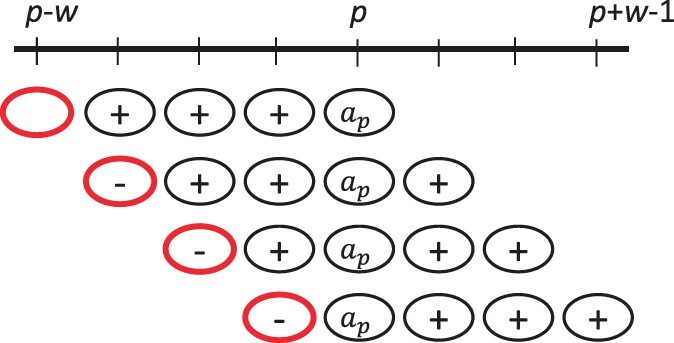
Illustration of charging. Each row shows a possible way that position *p* can charge an index, with *w *=* *4. A minus sign indicates the value is less than *a_p_*, a plus sign indicates the value is larger than *a_p_* and no sign indicates that it does not matter. The circle at the index that is charged is shown in bold red. Note that no two rows are compatible with each other, i.e. every row pair contains a column with both a plus and a minus. As a result, the index that gets charged is unique. (A color version of this figure appears in the online version of this article.)

Here, we use 1 in as an indicator function, i.e. $1(A_{p}=B_{q})$ is 1 if *A_p_* = *B_q_* and 0 otherwise. Next, we use the notion of a charged window from (Marçais *et al.*, 2017; Schleimer *et al.*, 2003). Given a position p∈[0,L−1] we say that *p charges* an index *i* if $i\in [\max\{-1,p-w\},p-1],\;a_{p}=\min\{a_{i+1},\ldots ,a_{\min(L-w-1,i+w)}\}$ and either i=max{−1,p−w} or *a_i_* < *a_p_*. Figure 2 illustrates the definition. For p∈[0,L−1] and i∈[−1,L−w−1] we define $X_{i,p}^{A}$ as an indicator random variable for the event that index *i* is charged by position *p*.

The following fact was already shown in Schleimer *et al.* (2003) and states that a minimizer charges exactly one window; Figure 2 shows the intuition behind it.

Fact 1. *Let* p∈[0,L−1]*. Position p is a minimizer in A iff there exists a unique* i∈[−1,L−w−1]  *such that p charges index i. In other words*, $M_{p}^{A}=\sum_{i=-1}^{L-w-1}X_{i,p}^{A}$.

Let us assume for the sake of simplicity and for this section only that *A* and *B* are padded. This allows us to combine Equation (1) with Fact 1 while avoiding edge cases and get:

$$\hat{I}=\sum_{i=0}^{L-w-1}\sum_{j=0}^{L-w-1}\sum_{p=i+1}^{i+w}\sum_{q=j+1}^{j+w}X_{i,p}^{A}X_{j,q}^{B}1(A_{p}=B_{q})$$

Applying linearity of expectation, the law of total probability, and the uniformity of the hash value distribution, we can show that

(2) $$E[\hat{I}]=\sum_{i=0}^{L-w-1}\sum_{j=0}^{L-w-1}\sum_{p=i+1}^{i+w}\sum_{q=j+1}^{j+w}\int_{0}^{1}Fdx,$$

where

$$F=\mathrm{Pr}[X_{i,p}^{A}=1,X_{j,q}^{B}=1|a_{p}=b_{q}=x]1(A_{p}=B_{q}).$$

To derive the value of the probability term *F*, let us fix *p* and *q* such that *A_p_* = *B_q_* and fix *a_p_* and *b_q_* to be some value *x*. Observe that in order for $X_{i,p}^{A}$ and $X_{j,q}^{B}$ to both be one, there are certain positions that need to have a hash value less than *x* (which happens with probability *x* for each position) and certain positions that need to have a hash value more than *x* (which happens with probability 1−x for each position). The hash values are pairwise independent, unless the two positions are in the *k*-mer-matching; in that case, the hash values are forced to be identical. If $X_{i,p}^{A}X_{j,q}^{B}=1$ imply contradictory values for at least one position, then *F* is zero. Otherwise, let *α* be the number of hash values that need to be less than *x*, but counting matched pairs only once. Similarly, let *β* denote the number hash values that need to be more than *x*, counting the matched pairs only once. Then,

$$\mathrm{Pr}[X_{i,p}^{A}X_{j,q}^{B}=1|a_{p}=b_{q}=x]=x^{\alpha }{\left(1-x\right)}^{\beta };$$

**Fig. 3:**
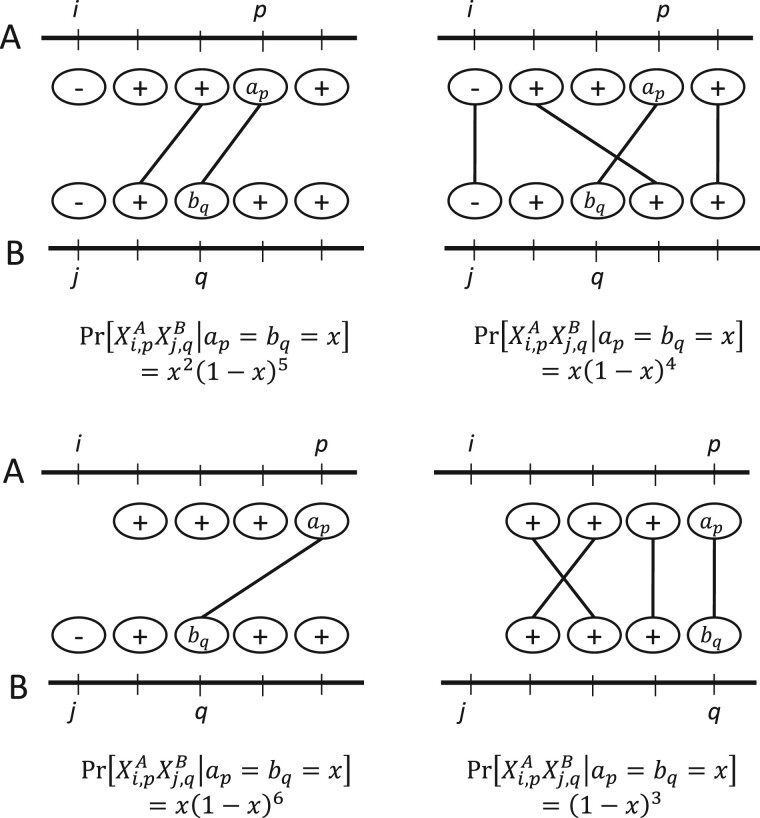
Some examples of $\mathrm{Pr}[X_{i,p}^{A}X_{j,q}^{B}=1|a_{p}=b_{q}=x]$, with *w *=* *4. The two horizontal lines correspond to sequences *A* and *B*, and a circle corresponds to a *k*-mer whose value is relevant to the probability. The lines between *A* and *B* show the *k*-mer-matching, i.e. they indicate that the corresponding *k*-mers are the same. A plus or minus sign at a position reflects that the hash value must be greater or less than *x*, respectively.

Figure 3 gives some examples.

Observe that 0≤α≤2 and 0≤β≤2(w−1). Therefore, the number of distinct terms in the summation of Eq. 2 is at most 6(w−1). The number of times each term is included in the summation is the number of i,j,p,q that induce the corresponding values of *α* and *β*. In Supplementary Appendix A.1, we formalize this notion using *configuration counts*; but, for the purposes of intuition, it suffices to observe that Equation (2) reduces to a function of the *k*-mer-matching, *w*, and *L*. We call this function C(A,B;w) and then obtain Lemma 1.

### 4.2 Lemma 2

In this section, we will prove Lemma 2, though we defer the proofs of the building blocks to the Supplementary Appendix. Lemma 1 gives a tight approximation of $E[\hat{I}]$ in terms of C. Now, we need to do the same for $E[\hat{U}]$.

Lemma 3.

$$\frac{4L}{w+1}-C(A,B;w)-10\leq E[\hat{U}(A,B;w)]\leq \frac{4L}{w+1}-C(A,B;w).$$

Now, with Lemmas 1 and 3, we can approximate $\frac{E[\hat{I}]}{E[\hat{U}]}$. The next step is to show that this ratio of expectations is a good approximation for the expectation of the ratio $\frac{\hat{I}}{\hat{U}}$, since $\hat{J}=\frac{\hat{I}}{\hat{U}}$. For this, we require asymptotically tight bounds on the variances of the random variables $\hat{I}$ and $\hat{U}$.

Lemma 4.

-
$\mathrm{Var}(\hat{I}(A,B;w))\leq 8w^{2}I(A,B)$ *;*
-
$\mathrm{Var}(\hat{U}(A,B;w))\leq 32w^{2}L$ .

By isolating the central part of the distributions and bounding the effect of the tails using Chebyshev’s inequality (Mitzenmacher and Upfal, 2017), we then obtain the following approximation for $E[\frac{\hat{I}}{\hat{U}}]$.

Lemma 5. $\left|E\left[\frac{\hat{I}}{\hat{U}}\right]-\frac{E[\hat{I}]}{E[\hat{U}]}\right|\leq \frac{11w^{2}}{\sqrt[{3\;}]{L}}.$

We now have the components to prove Lemma 2.

Proof (Lemma 2). For the lower bound, we note that

$E[\hat{J}]=E[\frac{\hat{I}}{\hat{U}}]\geq \frac{E[\hat{I}]}{E[\hat{U}]}-\frac{11w^{2}}{\sqrt[{3\;}]{L}}$ (Lemma 5)

$\geq \frac{C}{\frac{4L}{w+1}-C}-\frac{11w^{2}}{\sqrt[{3\;}]{L}}\;$ (Lemmas 1 and 3)

as claimed. For the upper bound, from Lemma 5, we know that

$$E[\hat{J}]=E[\frac{\hat{I}}{\hat{U}}]\leq \frac{E[\hat{I}]}{E[\hat{U}]}+\frac{11w^{2}}{\sqrt[{3}]{L}}.$$

The bounds from Lemmas 1 and 3 imply

$$E[\hat{J}]\leq \frac{C+2}{\frac{4L}{w+1}-C-10}+\frac{11w^{2}}{\sqrt[{3}]{L}}.$$

To complete the proof, we require two additional (and straightforward) bounds.

Fact 2. $C(A,B;w)\leq \frac{2L}{w+1}$.

Fact 3. *For all y > 20 and* 0<x≤y/2, $\frac{x+2}{y-x-10}-\frac{x}{y-x}\leq \frac{12}{y-5}$.

Letting x=C and $y=\frac{4L}{w+1}$, we have 0<x≤y/2 and *y *>* *20 (since L≥7(w+1)) and so

$$\begin{array}{c} E[\hat{J}]\leq \frac{C}{\frac{4L}{w+1}-C}+\frac{12}{\frac{4L}{w+1}-5}+\frac{11w^{2}}{\sqrt[{3}]{L}}\\ =\frac{C}{\frac{4L}{w+1}-C}+\frac{3(w+1)}{L-\frac{5(w+1)}{4}}+\frac{11w^{2}}{\sqrt[{3}]{L}}. \end{array}$$

Plugging in w+1≤L/7 and then using the fact that w≥2, we get

$$\begin{array}{c} E[\hat{J}]-J(A,B)\leq \frac{C}{\frac{4L}{w+1}-C}+\frac{84(w+1)}{23L}+\frac{11w^{2}}{\sqrt[{3}]{L}}\\ \leq \frac{C}{\frac{4L}{w+1}-C}+\frac{15w^{2}}{\sqrt[{3}]{L}}. \end{array}$$

□

## 5 Empirical results

### 5.1 Experimental setup

We use two different models to generate sequence pairs. In the *unrelated pair* model, we take a desired Jaccard value *j*, set $L=\frac{2j4^{k}}{j+1}$, and independently and randomly generate two duplicate-free strings *A* and *B* with *L k*-mers. We chose *L* in this way so that under the assumption that *A* and *B* are uniformly chosen, *j* is the expected value of *J*(*A*, *B*), over the randomness of the generative process. While such string pairs are unlikely to occur in practice for higher values of *j*, they allow us to observe the bias of unrelated pairs for whole range of Jaccard similarities. In the *related pair* model, *A* is a randomly selected substring of *Escherichia coli*  EColi download link (2021) with *L k*-mers. String *B* is created by sweeping along *A*, at each position deciding with probability *r*_1_ whether to mutate and then choosing a new nucleotide from those that would not create a duplicate *k*-mer. More details about the handling of special cases are in Supplementary Appendix A.6. Note that in both models, the generated sequences are not necessarily padded.

For each model, we generated 50 *hash replicates* hash function (unless otherwise noted) where each replicate uses a different seed for the hash function. We then report $\bar{J}$, which is the average of $\hat{J}$ over the hash replicates and is the empirical equivalent of $E[\hat{J}]$. We used the hash function that is part of minimap2 (Li, 2016), since the idealized hash function we assumed for the convenience of our theoretical proofs is not practical in software. For the mutation model, we also generated some number of *mutation replicates*, where each replicate is the result of re-running the random mutation process. In any experiment, the same set of hash seeds were used for every mutation replicate. Scripts to reproduce our experiments are available on our GitHub Paper Repo.

### 5.2 The extent of the empirical bias on real sequences

**Fig. 4:**
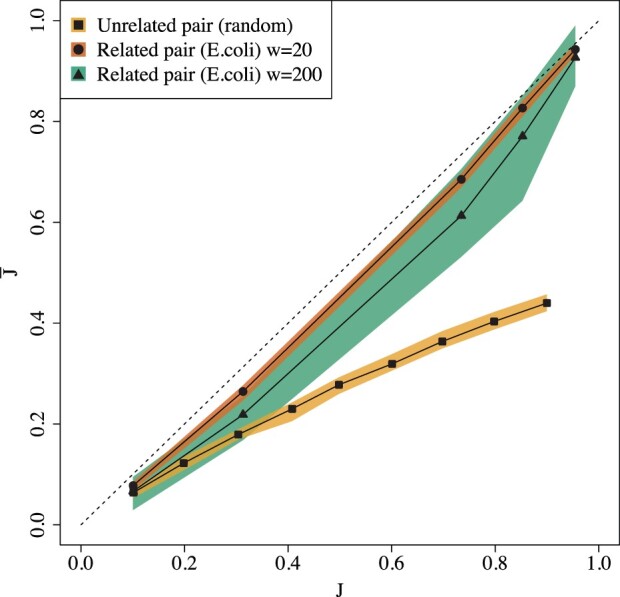
Empirical bias for unrelated and related sequence pairs. For the unrelated pairs, we used *w *=* *20 and *k *=* *8 for J≥.4 and *k *=* *7 for J≤.3. For related pairs, we set *k *=* *16, w∈{20,200}, *L *=* *10 000, and $r_{1}\in \{.001,.005,.01,.05,.1\}$, with one mutation replicate. The colored bands show the 2.5th and the 97.5th percentiles. The dashed line shows the expected behavior of an unbiased estimator, with $\bar{J}=J$.

Figure 4 shows that there is considerable bias across a wide range of Jaccard values, for both related and unrelated sequence pairs. There are pairs of sequences with a dramatic bias, e.g. for unrelated pair with a Jaccard of 90%, the estimator gives only 44%. In more practically relevant cases, the bias can remain substantial; e.g. when the true Jaccard of related pairs is 76%, the estimator gives only 65% (when *w *=* *200). The extent to which this bias is detrimental to the biological interpretation of the result depends on the downstream application. For example, using $\hat{J}$ to estimate the average nucleotide identity in order to build phylogenies, in the style of Mash (Ondov *et al.*, 2016), may be inadvisable.

**Fig. 5:**
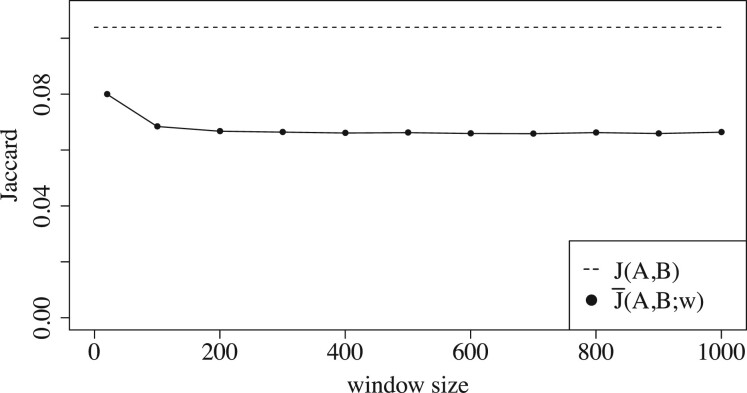
The effect of *w* on the empirical bias for a pair of related sequences as a function of the window size. Here, $r_{1}=0.1$, *L *=* *10 000, *k *=* *16, w∈{20,100,200,…,1000}, and there are 50 mutation replicates.

Figures 4 and 5 show the extent to which the empirical bias depends on the window size *w*. Figure 4 shows that the bias for related pairs can be twice as large for *w *=* *200 compared to *w *=* *20. Figure 5 gives a more fine-grained picture and shows how the absolute bias for a related sequence pair increases with *w*. We note that it plateaus for larger values of *w*.

**Fig. 6:**
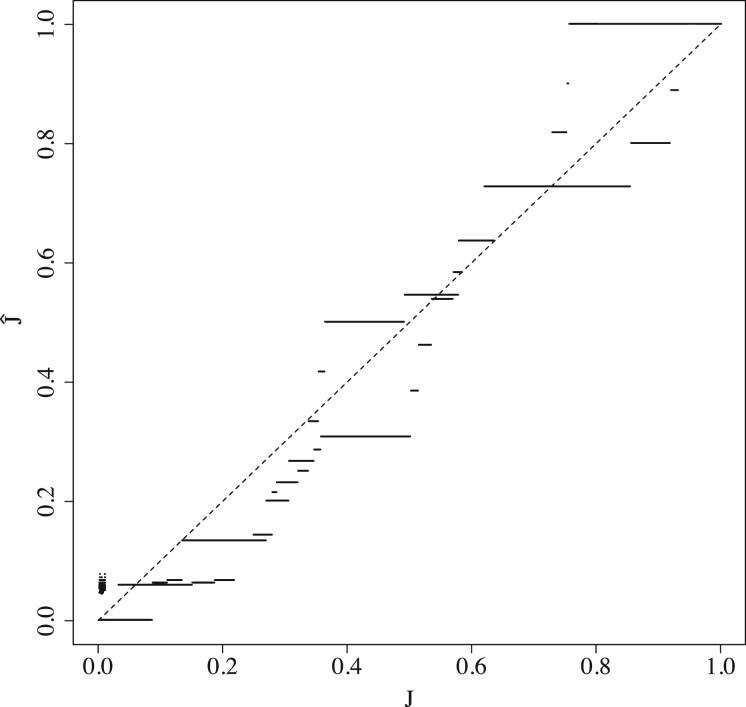
The empirical bias that occurs during a mapping process. Each point represents a comparison of a read *A* against a putative mapping location *B*. Note that the points visually blur into lines. We used *k *=* *16 and window size *w *=* *200 to match the default of mashmap. One hash replicate was used.

We also wanted to understand the extent of the bias in a scenario where the sequences are being compared as part of a read mapping process. To that end, we mimicked the behavior of the mashmap mapper (Jain *et al.*, 2017, 2018a) by taking one arbitrary substring *A* from hg38 chromosome 20, with *L *=* *1000, and comparing it against all substrings *B* with *L *=* *1000. Figure 6 show that during the alignment process, we encounter the whole range of true Jaccard values, and, for each one, there is a substantial but not drastic bias in $\hat{J}$. Unlike the prediction of Theorem 2, the bias is sometimes positive; after further investigation, this happens because the *A* and *B* in this experiment are not always padded, which is a condition of Theorem 2.

### 5.3 Effect of bias on mashmap sequence identity estimates

Mashmap is a read mapper that, for each mapped location, uses the Mash formula (Ondov *et al.*, 2016) to estimate the divergence (i.e. one minus the sequence identity) from $\hat{J}$. It was previously reported that the Mash formula’s use of a Poisson approximation makes it inaccurate for higher divergence (Ondov *et al.*, 2019; Sarmashghi *et al.*, 2019), so before proceeding further, we modified mashmap to replace this approximation with the exact Binomial-based derivation (we derive the correction formula in Supplementary Appendix A.6). We then simulated reads from *E.coli* with substitution errors to achieve a controlled divergence and mapped them back to the *E.coli* reference with mashmap (see Supplementary Appendix A.6 for more details). We used *k *=* *16 and mashmap automatically chose *w *=* *200 as the window size.

Table 1 shows that even after our correction, the mashmap divergence had an error, e.g. for a true divergence of 5.00%, mashmap reported an average divergence of 5.71%—an error of 14%. To confirm that this remaining error was due to the minimizer sketch, we replaced the $\hat{J}$ estimator in mashmap with the true Jaccard. Table 1 shows that after this replacement, the remaining error was reduced by an order of magnitude, e.g. mashmap now reported an average divergence of 4.99%. We therefore conclude that the bias we observe in mashmap after the Binomial correction is dominated by the bias of $\hat{J}$. In absolute terms, the $\hat{J}$ bias (about half a percentage point of divergence) may be acceptable for applications such as read alignment. However, for other applications (e.g. a fine grained analysis of sequence divergence), this bias may lead to downstream problems.

**Table 1.** The median sequence divergence reported by mashmap, over 100 trials, for unmodified mashmap (first row), mashmap after Binomial-correction (second row) and, in addition, the removal of the $\hat{J}$ bias

Mashmap estimator	True divergence		
	10.00	5.00	1.00
Unmodified	11.07	5.88	1.42
Corrected	10.48	5.71	1.41
Corrected + unbiased	10.05	4.99	1.00

### 5.4 Empirical accuracy of our B formula (Equation (3))

Theorem 1 predicts that our formula for B (Equation (3)) approximates the empirical bias. To empirically evaluate the quality of this approximation, we measured the empirical error of Equation (3), which we define to be the absolute difference between the empirically observed bias ($\bar{J}-J$) and B. For the sequence pairs used in Figure 4, the empirical error is never more than 0.007 and roughly one to two orders of magnitude smaller than the bias itself (Tables 2 and 3). This held across three hash function families we tested: the one used by minimap2 (Li, 2016), Murmurhash3 Murmurhash, and SplitMix64 (Steele *et al.*, 2014). Note that this robustness to different hash functions is not predicted by Theorem 1, which assumes an idealized version of a hash function which is collision free and maps uniformly to the real unit interval (in this case, none of the three functions map to the unit interval and Murmurhash3 is not collision free).

**Table 2.** The empirical error of our theoretically predicted bias (Equation (3)) on the related pair sequences of Figure 4

*r*_1_	0.001	0.005	0.010	0.050	0.100
*J*	0.10	0.27	0.74	0.90	0.99
B	−0.02	−0.05	−0.04	−0.02	−0.00
Error of B (mm2)	0.001	0.000	0.000	0.000	0.001
Error of B (mmh3)	0.001	0.000	0.001	0.001	0.000
Error of B (sm64)	0.000	0.000	0.002	0.000	0.000

*Note*: The error is measured with respect to three different hash function families: the minimap2 hash function (mm2), the Murmurhash3 hash function (mmh3) and the SplitMix64 hash function (sm64).

**Table 3.** The empirical error of our theoretically predicted bias (Equation (3)) on the unrelated pair sequences of Figure 4

*J*	0.1	0.2	0.3	0.4	0.5	0.6	0.7	0.8	0.9
B	−0.04	−0.08	−0.13	−0.17	−0.22	−0.28	−0.33	−0.39	−0.45
mm2	0.001	0.002	0.001	0.005	0.001	0.004	0.002	0.004	0.007
mmh3	0.001	0.000	0.000	0.002	0.003	0.002	0.001	0.003	0.003
sm64	0.001	0.000	0.001	0.002	0.002	0.003	0.002	0.001	−0.003

*Note*: The error is measured with respect to three different hash function families: the minimap2 hash function (mm2), the Murmurhash3 hash function (mmh3) and the SplitMix64 hash function (sm64).

**Fig. 7:**
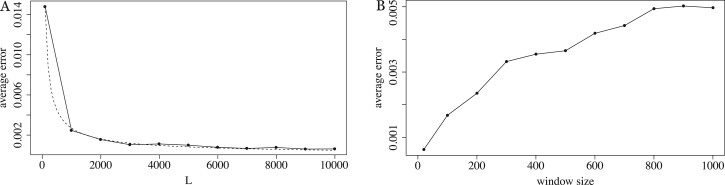
The effect of the window size *w* and sequence length on the empirical error of Equation (3). In panel **A**, we use the related pair model with 50 mutation replicates, *k *=* *16, *w *=* *20, $r_{1}=0.1$ and L∈{100,1000,2000,…,10000}. The *y*-axis shows the error of B, averaged over the mutation replicates. The dashed line shows the best fit function of the form $\alpha L^{\beta }$, computed using the nls function in R. The average *J*, over the mutation replicates, is between.101 and.106, and the average empirical bias ranged between –0.023 and –0.027, depending on *L*. In panel **B**, we use the related pair model with 50 mutation replicates, *k *=* *16, *L *=* *10 000, and w∈{20,100,200,…,1000}. The average *J* is.104.

We measured the effect of increasing *w* and decreasing *L* on the empirical error for a related pair (Fig. 7). The empirical error increases with *w* but remains almost two orders of magnitude smaller than the true Jaccard. For L≥1000, the empirical error is less than half a percent of the true Jaccard. Even for the smallest value of *L* (i.e. 100), the empirical error is only 2.6% of the true Jaccard. We conclude that Equation (3) is a high quality approximation for the empirically observed bias.

### 5.5 Accuracy of the *ε* bound to the approximation to Equation (3)

Theorem 1 states that the expected error of Equation (3) is at most $\varepsilon =\frac{100w^{2}}{\sqrt[{3\;}]{L}}$. Since this is only an upper bound, we wanted to check the tightness with respect to *w* and to *L*. For *w *=* *20 and non-astronomical values of *L*, ε>1 and thus Theorem 1 gives no guarantee on the accuracy of the B term. Empirically, however, the error is small (Fig. 7A), indicating that, at least for related pairs, *ε* is likely not a tight bound. To understand if the dependence on *L* is accurate, we found the best fit of a function of the form $\alpha L^{\beta }$ to the observed error curve in Figure 7A. The best fit was $0.44L^{-0.74}$, which indicates that our dependence on *L* in *ε* is not tight. One possible way to achieve this may be to use tighter concentration bounds than Chebyshev’s inequality inside the proof of Lemma 5 (leveraging the limited dependency between the events of *k*-mers being minimizers). Furthermore, Figure 7B suggests that the true error may be sub-linear in *w*, while *ε* has a *w*^2^ dependence. Thus our empirical results indicate that *ε* could potentially be improved for related sequences, though it may still be tight in the worst-case.

## 6 Discussion

In this article, we showed that the minimizer Jaccard estimator suffers from bias and inconsistency, using both theoretical and empirical approaches. The bias can be drastic in some fairly artificial cases (i.e. unrelated sequences with high Jaccard) but remains substantial even on more realistically related pairs of sequences. Our theoretical results indicate that the bias cannot be removed by decreasing the window size (except for the pathological case when *w *=* *1, where effectively there is no sketching done). We showed how the bias manifests in the mashmap read mapper as error in the reported sequence divergence. A future direction would be to derive the expected value of the bias B in the simple mutation model of Blanca *et al.* (2021); if B reduces to a function of *w* without depending on the *k*-mer layout, then it could potentially be used to correct the bias in mashmap. Even if that were not possible, one could still use the estimator provided that an experimental evaluation determines that the observed bias is tolerable for the downstream application. On the other hand, the bias problems can be sidestepped altogether by using a similar but unbiased sketch, e.g. the modulo sketch (Schleimer *et al.*, 2003). Finally, we note that while we focus on bias in this paper, it is not the only theoretical property of importance for sketching; for example, there has been much exploration of different hash functions (DeBlasio *et al.*, 2019; Edgar, 2021; Frith *et al.*, 2020; Jain *et al.*, 2020; Marçais *et al.*, 2017, 2018; Sahlin, 2021; Zheng *et al.*, 2020) to reduce the density and/or to select *k*-mers that have desirable properties such as conservation or spread (Shaw and Yu, 2021).

Our results also relate to the minhash minimizer Jaccard estimator (${\hat{J}}_{\text{minhash}}$) described by Jain *et al.* (2017). In this variant, the set of *k*-mers in a minimizer sketch is further reduced by taking the *s* smallest values (i.e. their minhash sketch); the Jaccard estimator is then computed between these reduced sets. If the minhash sketch is taken using a different hash function than was used for computing minimizers, then the classical result of Broder (1997) implies that $E[{\hat{J}}_{\text{minhash}}]=E[\hat{J}]$. This estimator would therefore suffer from the same bias that we have shown in this paper. If, on the other hand, the same hash values are reused, then the result of Broder (1997) is not applicable, because it assumes that the hash values being selected are uniformly random; in our case, the hash values being selected in the minhash step have already ‘won the competition’ of being smallest in their window. Though we did not explore the bias of this variant of ${\hat{J}}_{\text{minhash}}$, it would seem surprising if the minhash step somehow magically unbiased $\hat{J}$.

## Funding

This material is based upon work supported by the National Science Foundation under Grants No. 2029170, 1453527, 1931531, 1356529.

*Conflict of Interest*: none declared.

## Supplementary Material

btac244_Supplementary_Data
