# Supplementary material for: The minimizer Jaccard estimator is biased and inconsistent
Source: Bioinformatics. 2022 Jun 27;38(Suppl 1):i169–76. doi: 10.1093/bioinformatics/btac244 (PMC9235516; doi:10.1093/bioinformatics/btac244)
Supplement: btac244_Supplementary_Data [file btac244_supplementary_data.pdf]

A Appendix for "The minimizer Jaccard estimator is biased and inconsistent" by Mahdi Belbasi, Antonio Blanca, Robert S. Harris, David Koslicki, and Paul Medvedev

In this appendix, we will prove the main theorems of the paper as well as provide experimental details to aid reproducibility.

A.1 Matching configurations and the definition of $\mathcal{C}(A, B; w)$ and $\mathcal{B}(A, B; w)$

In this section, we define the notion of matching configurations and then use them to define $\mathcal{C}(A, B; w)$ and $\mathcal{B}(A, B; w)$. As discussed in Section 3, the bias of $\widehat{J}$ depends on the layout of the shared k-mers along the sequence. It turns out that the aspects of their sharedness that contribute to the bias are captured by the amount and location of k-mers that are shared between windows $\{A_i, \ldots, A_{i+w}\}$ and $\{B_j, \ldots, B_{j+w}\}$, for any i and j.

Let us define $S(i, j, \ell) \triangleq |\{A_i, \ldots, A_{i+\ell-1}\} \cap \{B_j, \ldots, B_{j+\ell-1}\}|$, i.e. the number of shared k-mers in the windows of length ℓ starting at positions i and j in A and B, respectively. We then define a *matching configuration* as a 5-tuple, written as

$$[\![C_{\text{a,left}}, C_{\text{a,right}}; C_{\text{b,left}}, C_{\text{b,right}}; s]\!],$$

where $s \in \{0, \ldots, w\}$ and $C_{\text{a,left}}, C_{\text{a,right}}, C_{\text{b,left}}, C_{\text{b,right}} \in \{0, 1, 2\}$. We then say that an index pair (i, j) with $i, j \in [0, L-w-1]$ has configuration $[\![C_{\text{a,left}}, C_{\text{a,right}}; C_{\text{b,left}}, C_{\text{b,right}}; s]\!]$ if the windows $\{A_{i+1}, \ldots, A_{i+w}\}$ and $\{B_{j+1}, \ldots, B_{j+w}\}$ share s k-mers (i.e., $s = S(i+1, j+1, w)$) and

$$C_{\text{a,left}} = \begin{cases} 0 \text{ if } A_i = B_j, \\ 1 \text{ if } A_i \in \{B_{j+1}, \ldots, B_{j+w}\}, \\ 2 \text{ otherwise;} \end{cases} \qquad C_{\text{a,right}} = \begin{cases} 0 \text{ if } A_{i+w} = B_{j+w}, \\ 1 \text{ if } A_{i+w} \in \{B_{j+1}, \ldots, B_{j+w-1}\}, \\ 2 \text{ otherwise;} \end{cases}$$

$$C_{\text{b,left}} = \begin{cases} 0 \text{ if } B_j = A_i, \\ 1 \text{ if } B_j \in \{A_{i+1}, \ldots, A_{i+w}\}, \\ 2 \text{ otherwise;} \end{cases} \qquad C_{\text{b,right}} = \begin{cases} 0 \text{ if } B_{j+w} = A_{i+w}, \\ 1 \text{ if } B_{j+w} \in \{A_{i+1}, \ldots, A_{i+w-1}\}, \\ 2 \text{ otherwise.} \end{cases}$$

An index pair (i, j) has exactly one configuration, and not all configurations are possible; in particular, configurations where exactly one of $C_{\text{a,left}}$ or $C_{\text{b,left}}$ is zero, or exactly one of $C_{\text{b,right}}$ and $C_{\text{a,right}}$ is zero, are impossible. Figure S1 shows some examples of configurations. We may label configuration elements as sets (e.g. $C_{\text{a,left}} = \{0, 2\}$) to indicate all the configurations that can be formed using values from that set, except for impossible configurations. We use $*$ as shorthand for the set $\{0, 1, 2\}$ of all possible values. For example, $[\![*, 0; *, 0; s]\!]$ refers to the configurations $[\![0, 0; 0, 0; s]\!]$, $[\![1, 0; 1, 0; s]\!]$, $[\![2, 0; 1, 0; s]\!]$, $[\![1, 0; 2, 0; s]\!]$, $[\![2, 0; 2, 0; s]\!]$. For a configuration C we use $N(C)$ to denote the number of pairs (i, j) such that the configuration of (i, j) is C.

In order to define $\mathcal{B}(A, B; w)$, we define first the quantity $\mathcal{C}(A, B; w)$. Let $t_0 = \frac{1}{2w-s}$, $t_1 = \frac{1}{(2w-s)(2w-s+1)}$, and $t_2 = \frac{1}{(2w-s)(2w-s+1)(2w-s+2)}$.

$$\begin{aligned}
\mathcal{C}(A, B; w) \triangleq \textstyle\sum_{s=0}^{w} & t_0 N([\![1, 0; 1, 0; s]\!]) && + \; t_0 N([\![1, 0; 2, 0; s]\!]) && + \; t_0 N([\![2, 0; 1, 0; s]\!])) \\
& + t_1 N([\![2, \{1, 2\}; 1, 1; s]\!]) && + \; t_1 N([\![1, 1; 2, \{1, 2\}; s]\!]) && + \; 2w t_1 N([\![0, 0; 0, 0; s]\!]) \\
& + t_1 s N([\![0, 1; 0, 1; s]\!]) && + \; t_1 s N([\![0, 1; 0, 2; s]\!]) && + \; t_1 s N([\![0, 2; 0, 1; s]\!]) \\
& + t_1 s N([\![0, 2; 0, 2; s]\!]) && + \; 2 t_2 s N([\![2, 2; 2, 2; s]\!]) && + \; 4 t_2 w N([\![2, 1; 2, 1; s]\!]) \\
& + t_2 (s + 2w) N([\![2, 1; 2, 2; s]\!]) && + \; t_2 (s + 2w) N([\![2, 2; 2, 1; s]\!]) \\
& + t_2 (6w - s + (2w - s)^2) N([\![2, 0; 2, 0; s]\!])
\end{aligned}$$

In particular, $\mathcal{C}(A, B; w)$ is a linear combination of configuration counts, where each count is weighted by some function of its s value and w. We also define $\mathcal{D}(A, B; w) = \sum_{s=0}^{w} N([\![*, 0; *, 0; s]\!])$. The term $\mathcal{B}(A, B; w)$, which essentially determines the bias of the Jaccard estimator

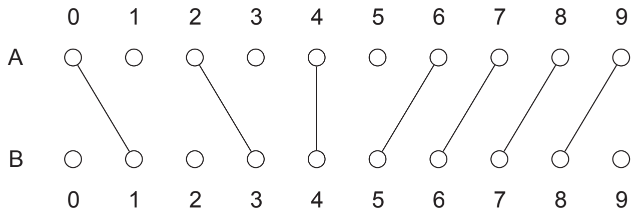

Fig. S1: Configuration examples with $w = 2$: the pair $(0, 1)$ has configuration $[\![0, 0; 0, 0; 1]\!]$; pair $(4, 4)$ has $[\![0, 1; 0, 2; 1]\!]$; pair $(7, 6)$ has $[\![0, 0; 0, 0; 2]\!]$.

(see Theorem 1), is defined as follows:

$$\mathcal{B}(A, B; w) \triangleq \frac{\mathcal{C}(A, B; w)}{\frac{4L}{w+1} - \mathcal{C}(A, B; w)} - \frac{\mathcal{D}(A, B; w)}{2L - \mathcal{D}(A, B; w)}. \quad (3)$$

A.2 Proof of Theorem 1

In all the following, we will assume that $L \geqslant 7(w+1)$.

A.2.1 Approximating the minimizer union and intersection (Lemmas 1 and 3)

In this section, we will prove Lemmas 1 and 3. First, we recapitulate the proof of Fact 1 in our notation:

Fact 1. *Let $p \in [0, L-1]$. Position p is a minimizer in A iff there exists a unique $i \in [-1, L-w-1]$ such that p charges index i. In other words, $M_p^A = \sum_{i=-1}^{L-w-1} X_{i,p}^A$.*

Proof. Figure 2 gives the intuition for the proof. For the only if direction, suppose that p charges index i. Then, by definition of charging, $a_p = \min\{a_{i+1}, \ldots, a_{i+w}\}$, and so p is a minimizer. For the if direction, suppose that p is a minimizer in A. Consider the leftmost window in which it is a minimizer, i.e. the smallest $i' \in [p-w+1, p]$ such that $a_p = \min\{a_{i'}, \ldots, a_{i'+w-1}\}$. Since i' is smallest, then either $i' = p-w+1$ or $a_{i'-1} < a_p$. This is the definition of p charging index $i'-1$. For uniqueness, consider all the possible windows that p can charge, shown in Figure 2. They are all pairwise incompatible, i.e. there is at least one position that is simultaneously required to be larger than a_p and smaller than a_p. □

The expected value of M_p^A is called the *density* of the minimizer scheme, and we compute it exactly in the following Fact. We note that similar derivations of the density also appeared in Schleimer et al. [2003], Roberts et al. [2004], but our proof accounts also for the edge cases.

Fact 4. *For $p \in [0, L-1]$, we have $\mathbb{E}[M_p^A] \leqslant \frac{2}{w+1}$. More precisely,*

$$\mathbb{E}[M_p^A] = \begin{cases} \frac{2}{w+1} & \text{for } p \in [w, L-w]; \\ \frac{w+1+p}{w(w+1)} & \text{for } p \in [0, w-1]; \\ \frac{L-p+w}{w(w+1)} & \text{for } p \in [L-w+1, L-1]. \end{cases}$$

Proof. Let $\ell = \max(-1, p-w)$ and $u = \min(L-w-1, p-1)$. For $i \in [\ell+1, u]$, we have $\Pr[X_{i,p}^A] = \int_0^1 \Pr[X_{i,p}^A \mid a_p = x]\, dx = \int_0^1 x(1-x)^{w-1}\, dx = \frac{1}{w(w+1)}$. For $i = \ell$, we have $\Pr[X_{i,p}^A] = \int_0^1 (1-x)^{w-1}\, dx = 1/w$.

By Fact 1, $M_p^A = \sum_{i=-1}^{L-w-1} X_{i,p}^A$. When $p \in [0, w-1]$, we have

$$M_p^A = X_{-1,p}^A + \sum_{i=0}^{p-1} X_{i,p}^A = \frac{1}{w} + \frac{p}{w(w+1)}.$$

When $p \in [w, L-w]$, we have

$$M_p^A = X_{p-w,p}^A + \sum_{i=p-w+1}^{p-1} X_{i,p}^A = \frac{1}{w} + \frac{w-1}{w(w+1)} = \frac{2}{w+1}.$$

When $p \in [L-w+1, L-1]$, we have

$$M_p^A = X_{p-w,p}^A + \sum_{i=p-w+1}^{L-w-1} X_{i,p}^A = \frac{1}{w} + \frac{L-p-1}{w(w+1)} = \frac{L-p+w}{w(w+1)}.$$

□

We are now ready to prove Lemma 1.

Lemma 1. $\mathcal{C}(A, B; w) \leqslant \mathbb{E}[\widehat{I}(A, B; w)] \leqslant \mathcal{C}(A, B; w) + 2$.

Proof. From the definition of $\widehat{I}(A, B; w)$ and Fact 1, we have

$$\widehat{I}(A, B; w) = \sum_{i=-1}^{L-w-1} \sum_{p=0}^{L-1} \sum_{j=-1}^{L-w-1} \sum_{q=0}^{L-1} X^A_{i,p} X^B_{j,q} \, \mathbb{1}(A_p = B_q).$$

Observe that by definition of charging, $X^A_{i,p} = 0$ when $p \notin [i+1, i+w]$. Therefore,

$$\widehat{I}(A, B; w) = \sum_{i=-1}^{L-w-1} \sum_{p=i+1}^{i+w} \sum_{j=-1}^{L-w-1} \sum_{q=j+1}^{j+w} X^A_{i,p} X^B_{j,q} \, \mathbb{1}(A_p = B_q).$$

We can ignore some of the boundary terms associated with position -1 being charged without much loss in accuracy. Let

$$\widehat{I}_{\text{core}} = \sum_{i=0}^{L-w-1} \sum_{p=i+1}^{i+w} \sum_{j=0}^{L-w-1} \sum_{q=j+1}^{i+w} X^A_{i,p} X^B_{j,q} \, \mathbb{1}(A_p = B_q).$$

We claim that $\mathbb{E}[\widehat{I}_{\text{core}}] \leqslant \mathbb{E}[\widehat{I}(A, B; w)] \leqslant \mathbb{E}[\widehat{I}_{\text{core}}] + 2$. The lower bound is immediate. For the upper bound, let us first separate out the terms of $\widehat{I}$ with $i = -1$ or $j = -1$:

$$\widehat{I}(A, B; w) \leqslant \widehat{I}_{\text{core}} + \sum_{i=-1}^{L-w-1} \sum_{p=i+1}^{i+w} \sum_{q=0}^{w-1} X^A_{i,p} X^B_{-1,q} \, \mathbb{1}(A_p = B_q) + \sum_{p=0}^{w-1} \sum_{j=-1}^{L-w-1} \sum_{q=j+1}^{j+w} X^A_{-1,p} X^B_{j,q} \, \mathbb{1}(A_p = B_q)$$

For the second term, observe that, by definition of charging, there is at most one value of q for which $X^B_{-1,q} = 1$. Then, since there are no repeated k-mers in A or B, there is at most one value of p for which $A_p = B_q$. Finally, by definition of charging, there is at most one value of i for which $X^A_{i,p} = 1$. Hence the second term is at most one; by a symmetrical argument, the third term is at most one as well. This gives us the desired upper bound.

It now suffices to show that $\mathbb{E}[\widehat{I}_{\text{core}}] = \mathcal{C}(A, B; w)$.

$$\begin{aligned}
\mathbb{E}[\widehat{I}_{\text{core}}] &= \sum_{i=0}^{L-w-1} \sum_{j=0}^{L-w-1} \sum_{p=i+1}^{i+w} \sum_{q=j+1}^{j+w} \mathbb{E}[X^A_{i,p} X^B_{j,q}] \, \mathbb{1}(A_p = B_q) \\
&= \sum_{i=0}^{L-w-1} \sum_{j=0}^{L-w-1} \sum_{p=i+1}^{i+w} \sum_{q=j+1}^{j+w} \Pr[X^A_{i,p} = 1, X^B_{j,q} = 1] \, \mathbb{1}(A_p = B_q) \\
&= \sum_{i=0}^{L-w-1} \sum_{j=0}^{L-w-1} \sum_{p=i+1}^{i+w} \sum_{q=j+1}^{j+w} \int_0^1 \Pr[X^A_{i,p} = 1, X^B_{j,q} = 1 \mid a_p = b_q = x] \, \mathbb{1}(A_p = B_q) \, dx.
\end{aligned}$$

The probability $\Pr[X^B_{j,q} = 1, X^A_{i,p} = 1 \mid a_p = b_q = x]$ will depend on the configuration of the indices i and j and on whether $p = i + w$ or $q = j + w$. Therefore, we rearrange the sums as follows. For a configuration c, we say that $(i, j) \to c$ when the indices i and j are in configuration c, so that

$$\begin{aligned}
\mathbb{E}[\widehat{I}_{\text{core}}] =& \sum_c \sum_{(i,j)\to c} \sum_{p=i+1}^{i+w} \sum_{q=j+1}^{j+w} \int_0^1 \Pr[X^A_{i,p} = 1, X^B_{j,q} = 1 \mid a_p = b_q = x] \, \mathbb{1}(A_p = B_q) \, dx \\
=& \sum_c \sum_{(i,j)\to c} \sum_{p=i+1}^{i+w-1} \sum_{q=j+1}^{j+w-1} \int_0^1 \Pr[X^A_{i,p} = 1, X^B_{j,q} = 1 \mid a_p = b_q = x] \, \mathbb{1}(A_p = B_q) \, dx \qquad (4) \\
&+ \sum_c \sum_{(i,j)\to c} \sum_{p=i+1}^{i+w} \int_0^1 \Pr[X^A_{i,p} = 1, X^B_{j,j+w} = 1 \mid a_p = b_{j+w} = x] \, \mathbb{1}(A_p = B_q) \, dx \qquad (5) \\
&+ \sum_c \sum_{(i,j)\to c} \sum_{q=j+1}^{j+w-1} \int_0^1 \Pr[X^A_{i,i+w} = 1, X^B_{j,q} = 1 \mid a_{i+w} = b_q = x] \, \mathbb{1}(A_p = B_q) \, dx. \qquad (6)
\end{aligned}$$

Figure 3 gives some examples to develop the intuition for what the inner term can evaluate to. We consider next each summation Equation (4), Equation (5), and Equation (6) separately. We start with Equation (5). Note that in this case the value of q is fixed to $j + w$, and so there is at most one value of p in the summation that is not 0 (since $A_p = B_q$). We partition the space of all configurations into four possible cases: (i) $c = [\![*, 0; *, 0; s]\!]$, (ii) $[\![\{0, 2\}, *; *, 1; s]\!]$, (iii) $c = [\![1, *; *, 1; s]\!]$, and (iv) $c = [\![*, *; *, 2; s]\!]$.

First note that for any c, we have $X^B_{j,j+w} = 1$ if and only if $b_{j+1}, \ldots, b_{j+w-1}$ are each greater than x. In case (i) when $c = [\![*, 0; *, 0; s]\!]$, the only value of p for which the probability in Equation (5) is not zero is $p = i + w$. From the definition of charging, we have $X^A_{i,i+w} = 1$ and

$X^B_{j,j+w} = 1$ if and only if $a_{i+1}, \dots, a_{i+w-1}, b_{j+1}, \dots, b_{j+w-1}$ are each greater than x. The number of distinct k-mers in this sequence is $2w - 2 - S(i+1, j+1, w-1) = 2w - 2 - S(i+1, j+1, w) + 1 = 2w - 1 - s$. Therefore, $\Pr[X^A_{i,p} = 1, X^B_{j,j+w} = 1 \mid a_p = b_q = x] = (1-x)^{2w-1-s}$ and

$$\sum_{p=i+1}^{i+w} \int_0^1 \Pr[X^A_{i,p} = 1, X^B_{j,j+w} = 1 \mid a_p = b_{j+w} = x] \, \mathbb{1}(A_p = B_{w+j}) \, dx = \int_0^1 (1-x)^{2w-1-s} \, dx = t_0,$$

recalling that $t_0 = \frac{1}{2w-s}$, $t_1 = \frac{1}{(2w-s)(2w-s+1)}$, and $t_2 = \frac{1}{(2w-s)(2w-s+1)(2w-s+2)}$. For case (ii) with $c = [\![\{0,2\}, *; *, 1; s]\!]$, because $C_{\text{b,right}} = 1$, the only value of p for which the probability in Equation (5) is not zero belongs to $[i+1, i+w-1]$. From the definition of charging, we have $X^A_{i,p} = 1$ iff $a_i < x$ and $a_{i+1}, \dots, a_{i+w}$, with the exception of a_p, are all greater than x. As mentioned previously, we have that $X^B_{j,j+w} = 1$ iff $b_{j+1}, \dots, b_{j+w-1}$ are each greater than x. Because $C_{\text{a,left}} \neq 1$, we have $A_i \notin \{B_{j+1}, \dots, B_{j+w-1}\}$. Therefore, we have one hash value (i.e. a_i) that is less than x, and $2w - 2 - (S(i+1, j+1, w) - 1)$ distinct hash values that are more than x. As a result,

$$\sum_{p=i+1}^{i+w} \int_0^1 \Pr[X^A_{i,p} = 1, X^B_{j,j+w} = 1 \mid a_p = b_{j+w} = x] \, \mathbb{1}(A_p = B_{j+w}) \, dx = \int_0^1 x(1-x)^{2w-1-s} \, dx \qquad = t_1.$$

For next two cases (i.e., case (iii) and (iv)) we show that the sum is 0. When $c = [\![1, *; *, 1; s]\!]$, the fact that $C_{\text{b,right}} = 1$ means that $C_{\text{a,right}} \neq 0$ which implies that $p < i + w$ and that, if $X^A_{i,p} = 1$, then $a_i < x$. The fact that $C_{\text{a,left}} = 1$ implies that $A_i \in \{B_{j+1}, \dots, B_{j+w}\}$. Therefore, one of the values of $\{b_{j+1}, \dots, b_{j+w}\}$ is less than x, which makes it impossible that $X^B_{j,q} = 1$. When $c = [\![*, *; *, 2; s]\!]$, there is no value of $p \in [i+1, i+w]$ which satisfies $A_p = B_{j+w}$, so $\mathbb{1}(A_p = B_{j+w}) = 0$. Putting all the four cases together, we have shown that the inner summation in Equation (5) is:

$$\begin{aligned} \sum_c \sum_{(i,j)\to c} \sum_{p=i+1}^{i+w} \int_0^1 & \Pr[X^A_{i,p} = 1, X^B_{j,j+w} = 1 \mid a_p = b_{j+w} = x] \, \mathbb{1}(A_p = B_q) \, dx \\ &= \sum_{s=0}^{w} t_0 N([\![*, 0; *, 0; s]\!]) + t_1 N([\![\{0,2\}, *; *, 1; s]\!]). \end{aligned} \tag{7}$$

Deriving a closed form for Equation (6) is symmetric to Equation (5) with the exception that when $c = [\![*, 0; *, 0; s]\!]$, there is no value of q in the range of the sum (i.e. $q \in [j+1, j+w-1]$) such that $A_{i+w} = B_q$. Hence, for the inner summation in Equation (6), we obtain

$$\begin{aligned} \sum_c \sum_{(i,j)\to c} \sum_{q=j+1}^{j+w-1} \int_0^1 & \Pr[X^A_{i,i+w} = 1, X^B_{j,q} = 1 \mid a_{i+w} = b_q = x] \, \mathbb{1}(A_p = B_q) \, dx \\ &= \sum_{s=0}^{w} t_1 N([\![*, 1; \{0,2\}, *; s]\!]) \end{aligned} \tag{8}$$

With a similar but more delicate case-by-case analysis, we also derive a closed form for Equation (4), whose proof we postpone until later.

Fact 5. *Let*

$$T = \sum_c \sum_{(i,j)\to c} \sum_{p=i+1}^{i+w-1} \sum_{q=j+1}^{j+w-1} \int_0^1 \Pr[X^A_{i,p} = 1, X^B_{j,q} = 1 \mid a_p = b_q = x] \, \mathbb{1}(A_p = B_q) \, dx.$$

Then,

$$\begin{aligned} T = \textstyle\sum_{s=0}^{w} & s t_1 N([\![0, 2; 0, 2; s]\!]) + 2 s t_2 N([\![2, 2; 2, 2; s]\!]) + 2(s-2) t_2 N([\![2, 1; 2, 1; s]\!]) \\ &+ (s-2) t_1 N([\![0, 1; 0, 1; s]\!]) + (s-1) t_1 (N([\![0, 1; 0, 2; s]\!]) + N([\![0, 2; 0, 1; s]\!]) + N([\![0, 0; 0, 0; s]\!])) \\ &+ 2(s-1) t_2 (N([\![2, 1; 2, 2; s]\!]) + N([\![2, 2; 2, 1; s]\!]) + N([\![2, 0; 2, 0; s]\!])). \end{aligned} \tag{9}$$

Finally, observe that summing Equation (7), Equation (8) and Equation (9) and then collecting the coefficients for each configuration, we obtain that $\mathbb{E}[\widehat{I}_{\text{core}}] = \mathcal{C}(A, B; w)$ as desired. □

We proceed with the proof of Fact 5.

Proof of Fact 5. For ease of notation, for a configuration c and a pair $(i,j) \to c$, let

$$H(c, i, j) = \sum_{p=i+1}^{i+w-1} \sum_{q=j+1}^{j+w-1} \int_0^1 \Pr[X^A_{i,p} = 1, X^B_{j,q} = 1 \mid a_p = b_q = x] \, \mathbb{1}(A_p = B_q) \, dx.$$

Since $p \neq i + w$ and $q \neq j + w$, we have that $X^A_{i,p} = 1$ and $X^B_{j,q} = 1$ iff $a_i < x$, $b_j < x$, and $a_{i+1}, \dots, a_{i+w}, b_{j+1}, \dots, b_{j+w}$, with the exception of a_p and b_q, are each greater than x. This corresponds to $2w - 1 - s$ hash values needing to be greater than x. What remains is to compute how many hash values need to be less than x.

We will partition the space of configurations into four possible cases: $[\![0,*;0,*;s]\!]$, $[\![2,*;2,*;s]\!]$, $[\![*,*;1,*;s]\!]$, and $[\![1,*;*,*;s]\!]$. First, consider the case of $c = [\![0,*;0,*;s]\!]$. In this case, $A_i = B_j$. Therefore,

$$H\left([\![0,*;0,*;s]\!], i, j\right) = \sum_{p=i+1}^{i+w-1} \sum_{q=j+1}^{j+w-1} \int_0^1 x(1-x)^{2w-1-s}\, dx = \sum_{p=i+1}^{i+w-1} \sum_{q=j+1}^{j+w-1} t_1 = t_1 S(i+1, j+1, w-1).$$

Next, consider the case of $[\![2,*;2,*;s]\!]$. This case is exactly the same as $c = [\![0,*;0,*;s]\!]$, except that $A_i \neq B_j$ and so

$$H\left([\![2,*;2,*;s]\!], i, j\right) = \sum_{p=i+1}^{i+w-1} \sum_{q=j+1}^{j+w-1} \int_0^1 x^2(1-x)^{2w-1-s}\, dx = 2t_2 S(i+1, j+1, w-1)$$

Next, observe that

$$S(i+1, j+1, w-1) = s - \begin{cases} 0 \text{ if } C_{\text{a,right}} = 2 \text{ and } C_{\text{b,right}} = 2 \\ 1 \text{ if } C_{\text{a,right}} = 0 \text{ and } C_{\text{b,right}} = 0 \\ 1 \text{ if } C_{\text{a,right}} = 1 \text{ and } C_{\text{b,right}} = 2 \\ 1 \text{ if } C_{\text{a,right}} = 2 \text{ and } C_{\text{b,right}} = 1 \\ 2 \text{ if } C_{\text{a,right}} = 1 \text{ and } C_{\text{b,right}} = 1\ , \end{cases} \tag{10}$$

where recall that $s = S(i+1, j+1, w)$. Therefore,

$$\begin{aligned}
&H([\![0,2;0,2;s]\!], i, j) = st_1, \\
&H([\![0,1;0,2;s]\!], i, j) = H([\![0,2;0,1;s]\!], i, j) = H([\![0,0;0,0;s]\!], i, j) = (s-1)t_1, \\
&H([\![0,1;0,1;s]\!], i, j) = (s-2)t_1, \\
&H([\![2,2;2,2;s]\!], i, j) = 2st_2, \\
&H([\![2,1;2,2;s]\!], i, j) = H([\![2,2;2,1;s]\!], i, j) = H([\![2,0;2,0;s]\!], i, j) = 2(s-1)t_2, \\
&H([\![2,1;2,1;s]\!], i, j) = 2(s-2)t_2.
\end{aligned}$$

Now, when $c = [\![1,*;*,*;s]\!]$, $A_i \in \{B_{j+1}, \ldots, B_{j+w}\}$. However, we already argued that $a_i < x$ and that $b_{j+1}, \ldots, b_{j+w}$ are all at least x. Hence, we cannot have both $X^A_{i,p} = 1$ and $X^B_{j,q} = 1$, and this type of configuration does not contribute to the sum. The case of $c = [\![*,*;1,*;s]\!]$ is symmetric. Finally, observing that $T = \sum_c \sum_{(i,j)\to c} H(c, i, j)$, we combine all the cases to get the desired equality of the fact statement. □

We now restate Lemma 3, whose proof is a direct consequence of Lemma 1.

Lemma 3.
$$\frac{4L}{w+1} - \mathcal{C}(A, B; w) - 10 \leqslant \mathbb{E}[\widehat{U}(A, B; w)] \leqslant \frac{4L}{w+1} - \mathcal{C}(A, B; w).$$

Proof. Recall that M^A_p denotes the indicator random variable for A_p being a minimizer in A. Then

$$\mathbb{E}[\widehat{U}(A, B; w)] = \sum_{p=0}^{L-1} \mathbb{E}[M^A_p] + \sum_{q=0}^{L-1} \mathbb{E}[M^B_q] - \mathbb{E}[I(A, B; w)] = 2\sum_{p=0}^{L-1} \mathbb{E}[M^A_p] - \mathbb{E}[\widehat{I}(A, B; w)].$$

From Lemma 1, we know that $\mathbb{E}[\widehat{I}(A, B; w)] \geqslant \mathcal{C}(A, B; w)$, and from Fact 4 we get that $\sum_{p=0}^{L-1} \mathbb{E}[M^A_p] \leqslant \frac{2L}{w+1}$. Combining these two facts, we deduce

$$\mathbb{E}[\widehat{U}(A, B; w)] \leqslant \frac{4L}{w+1} - \mathcal{C}(A, B; w),$$

as desired. For the lower bound, from Fact 4 we can deduce that

$$\sum_{p=0}^{L-1} \mathbb{E}[M^A_p] \geqslant \sum_{p=w}^{L-w} \mathbb{E}[M^A_p] = \frac{2(L-2w+1)}{w+1} \geqslant \frac{2L}{w+1} - \frac{4w-2}{w+1} \geqslant \frac{2L}{w+1} - 4.$$

The lower bound then follows from Lemma 1. □

A.2.2 Approximating the ratio of the minimizer union and intersection (Lemmas 4 and 5)

We begin this section with the proof of Lemma 4, where we obtain bounds for the variances of $\widehat{I}(A, B; w)$ and $\widehat{U}(A, B; w)$.

Lemma 4.

(i) $Var(\widehat{I}(A, B; w)) \leqslant 8w^2\, I(A, B)$;

(ii) $Var(\widehat{U}(A, B; w)) \leqslant 32w^2\, L$.

Proof. For ease of notation, we let $I = I(A, B)$ and $U = U(A, B)$. If p is a position in A, then define $w_p = \{A_{\max\{0,p-w+1\}}, \ldots, A_{\min\{p+w-1,L-1\}}\}$ and, if $x = A_p$, we say that the k-mers in w_p are *nearby* x in A.

We begin with part (i). For ease of notation set $\widehat{I} = \widehat{I}(A, B; w)$ and recall that

$$\widehat{I} = \sum_{p=0}^{L-1}\sum_{q=0}^{L-1} M_p^A M_q^B\, \mathbb{1}(A_p = B_q).$$

Then,

$$\begin{aligned}\mathbb{E}[\widehat{I}^2] &= \mathbb{E}\left[\left(\sum_{p=0}^{L-1}\sum_{q=0}^{L-1} M_p^A M_q^B\, \mathbb{1}(A_p = B_q)\right)\left(\sum_{p'=0}^{L-1}\sum_{q'=0}^{L-1} M_{p'}^A M_{q'}^B\, \mathbb{1}(A_{p'} = B_{q'})\right)\right]\\ &= \sum_{p=0}^{L-1}\sum_{q=0}^{L-1}\sum_{p'=0}^{L-1}\sum_{q'=0}^{L-1} \mathbb{E}[M_p^A M_q^B M_{p'}^A M_{q'}^B]\, \mathbb{1}(A_p = B_q)\, \mathbb{1}(A_{p'} = B_{q'}).\end{aligned}$$

Observe that $M_p^A M_q^B$ and $M_{p'}^A M_{q'}^B$ are independent if $|p - p'| > 2(w-1)$, $|q - q'| > 2(w-1)$, $w_p \cap w_{q'} = \varnothing$, and $w_{p'} \cap w_q = \varnothing$, since these four conditions guarantee that the two windows of size $2w-1$ centered at p and q (which determine $M_p^A M_q^B$) do not share k-mers with the two windows centered of size $2w-1$ at p' and q' (which determine $M_{p'}^A M_{q'}^B$).

Let D bet the set of tuples (p, q, p', q') such that $p, q, p', q' \in [0, L)$, $A_p = B_q$, $A_{p'} = B_{q'}$ and at least one of the following conditions hold: (i) $|p - p'| \leqslant 2(w-1)$, (ii) $|q - q'| \leqslant 2(w-1)$, (iii) $w_p \cap w_{q'} \neq \varnothing$, or (iv) $w_{p'} \cap w_q \neq \varnothing$. That is, D contains all tuples (p, q, p', q') for which $M_p^A M_q^B$ and $M_{p'}^A M_{q'}^B$ could be dependent, so that

$$\mathbb{E}[\widehat{I}^2] \leqslant |D| + \left(\sum_{p=0}^{L-1}\sum_{q=0}^{L-1} \mathbb{E}[M_p^A M_q^B]\, \mathbb{1}(A_p = B_q)\right)\left(\sum_{p'=0}^{L-1}\sum_{q'=0}^{L-1} \mathbb{E}[M_{p'}^A M_{q'}^B]\, \mathbb{1}(A_{p'} = B_{q'})\right) = |D| + \mathbb{E}[\widehat{I}]^2.$$

Then, $Var(\widehat{I}) = \mathbb{E}[\widehat{I}^2] - \mathbb{E}[\widehat{I}]^2 \leqslant |D|$ and it thus suffices to derive an upper bound for $|D|$. To do so, we will count the number of tuples that satisfy each of the conditions on the definition of D and add them together together to get an upper bound on $|D|$. For condition (i), there are I values of (p, q) such that $A_p = B_q$, and for each one, there are $4w - 3$ possible values of p' such that $|p - p'| \leqslant 2(w-1)$. Then, for a given value of p', there is at most one value of q' that would satisfy $A_{p'} = B_{q'}$. Therefore there are at most $(4w-3)I$ values of (p, q, p', q') that satisfy condition (i), i.e. $A_p = B_q$, $A_{p'} = B_{q'}$ and $|p - p'| \leqslant 2(w-1)$. By the same logic, there are at most $(4w-3)I$ values of (p, q, p', q') that satisfy condition (ii), i.e. $A_p = B_q$, $A_{p'} = B_{q'}$ and $|q - q'| \leqslant 2(w-1)$.

For condition (iii), again there are I values of (p, q) such that $A_p = B_q$. Then, each k-mer $x \in w_p$ can occur at most once in B, hence there are at most $2w - 1$ values of q' such that $x \in w_{q'}$. Since $|w_p| = 2w - 1$, there are at most $(2w-1)^2$ values of q' such that $w_p \cap w_{q'} \neq \varnothing$. For each value of q', there is at most one value of p' such that $B_{q'} = A_{p'}$. Therefore, there are at most $I(2w-1)^2$ values of (p, q, p', q') that satisfy condition (iii), i.e. $A_p = B_q$, $A_{p'} = B_{q'}$ and $w_p \cap w_{q'} \neq \varnothing$. By symmetric logic, the number of tuples that satisfy condition (iv) is also $I(2w-1)^2$.

Putting this all together, we get $Var(\widehat{I}) \leqslant |D| \leqslant 2(4w - 3 + (2w-1)^2)I \leqslant 8w^2 I$, which completes the proof of part (i).

We prove part (ii) next. For a k-mer $x \in U$, let U_x be the indicator random variable for the event that $x \in \widehat{U}(A, B; w)$. Let D be the set of all (x, y) pairs such that $x \in U$, $y \in U$, and U_x and U_y are dependent. Then,

$$\mathbb{E}[\widehat{U}^2] = \mathbb{E}\left[\sum_{x\in U} U_x \sum_{y\in U} U_y\right] = \sum_{x\in U}\sum_{y\in U} \mathbb{E}[U_x U_y] \leqslant |D| + \sum_{x\in U}\sum_{y\in U} \mathbb{E}[U_x]\mathbb{E}[U_y] = |D| + \mathbb{E}[\widehat{U}]^2,$$

and $Var(\widehat{U}) = \mathbb{E}[\widehat{U}^2] - \mathbb{E}[\widehat{U}]^2 \leqslant |D|$. It thus suffices to derive an upper bound for $|D|$. Let x and y belong to U. If U_x and U_y are dependent, then at least one of the following holds:

(i) One of the sequences (i.e. either A or B) contains both x and y at a distance of at most $2(w-1)$.

(ii) A contains x, B contains y, and the nearby k-mers of x in A intersect with the nearby k-mers of y in B.

(iii) B contains x, A contains y, and the nearby k-mers of x in B intersect with the nearby k-mers of y in A.

We will count the possible number of (x, y) pairs that satisfy each of the conditions and use their sum as an upper bound on $|D|$. For (i), there are 2 choices for which sequence contains x and y, at most L choices for the position of x, and at most $4w - 3$ choices for the position of y. Hence, there

are at most $2L(4w-3)$ choices for x and y that satisfy (i). For (ii), there are at most L choices for the position of x. If y satisfies the condition, then there must exist a k-mer z which is nearby to x in A and also nearby to y in B. There are at most $4w-3$ choices for z, and, for each of those choices, there are at most $4w-3$ locations for y. Hence, there are at most $L(4w-3)^2$ choices for x and y that satisfy (ii). Case (iii) is symmetrical to case (ii). In total then, $|D| \leqslant 2L(4w-3) + 2L(4w-3)^2 \leqslant 32w^2L$. □

With these bounds for the variances of $\widehat{I}(A,B;w)$ and $\widehat{U}(A,B;w)$ we can now prove Lemma 5.

Lemma 5. $\left|\mathbb{E}\left[\frac{\widehat{I}}{\widehat{U}}\right] - \frac{\mathbb{E}[\widehat{I}]}{\mathbb{E}[\widehat{U}]}\right| \leqslant \frac{11w^2}{\sqrt[3]{L}}$.

Proof. We start by introducing some convenient notation. Let $c = \sqrt[6]{L}$, $\sigma_{\mathrm{i}} = \sqrt{Var(\widehat{I})}$ and $\sigma_{\mathrm{u}} = \sqrt{Var(\widehat{U})}$. We say that $\widehat{I}$ and $\widehat{U}$ are *good* if their values lie in the range $\mathbb{E}[\widehat{I}] \pm c\sigma_{\mathrm{i}}$ and $\mathbb{E}[\widehat{U}] \pm c\,\sigma_{\mathrm{u}}$, respectively; otherwise we say they are *bad*. Let $\widehat{R} = \widehat{I}/\widehat{U}$. Note that $\mathbb{E}[\widehat{R}] = T_1 + T_2$, where

$$T_1 = \mathbb{E}\left[\widehat{R} \mid \ \widehat{I} \text{ and } \widehat{U} \text{ are good}\right] \Pr[\widehat{I} \text{ and } \widehat{U} \text{ are good}],$$

$$T_2 = \mathbb{E}\left[\widehat{R} \mid \ \widehat{I} \text{ or } \widehat{U} \text{ are bad}\right] \Pr[\widehat{I} \text{ or } \widehat{U} \text{ are bad}].$$

We will bound T_1 and T_2 separately. Observe that by Chebyshev's inequality Mitzenmacher and Upfal [2017], the probability that $\widehat{I}$ is bad is at most c^{-2} and the same holds for $\widehat{U}$. Hence, a union bound implies that $\Pr[\widehat{I} \text{ or } \widehat{U} \text{ are bad}] \leqslant 2c^{-2}$. Since $\widehat{I} \leqslant \widehat{U}$, $\widehat{R} \leqslant 1$, and we obtain the following bounds for T_2:

$$0 \leqslant T_2 \leqslant \Pr\left[\widehat{I} \text{ or } \widehat{U} \text{ are bad}\right] \leqslant 2c^{-2}.$$

For T_1, observe that

$$\mathbb{E}\left[\widehat{R} \mid \ \widehat{I} \text{ and } \widehat{U} \text{ are good}\right] \leqslant \mathbb{E}\left[\frac{\mathbb{E}[\widehat{I}] + c\sigma_{\mathrm{i}}}{\mathbb{E}[\widehat{U}] - c\,\sigma_{\mathrm{u}}}\right] \leqslant \frac{\mathbb{E}[\widehat{I}] + c\sigma_{\mathrm{i}}}{\mathbb{E}[\widehat{U}] - c\,\sigma_{\mathrm{u}}},$$

$$\mathbb{E}\left[\widehat{R} \mid \ \widehat{I} \text{ and } \widehat{U} \text{ are good}\right] \geqslant \mathbb{E}\left[\frac{\mathbb{E}[\widehat{I}] - c\sigma_{\mathrm{i}}}{\mathbb{E}[\widehat{U}] + c\,\sigma_{\mathrm{u}}}\right] \geqslant \frac{\mathbb{E}[\widehat{I}] - c\sigma_{\mathrm{i}}}{\mathbb{E}[\widehat{U}] + c\,\sigma_{\mathrm{u}}}.$$

Also, since $\Pr[\widehat{I} \text{ or } \widehat{U} \text{ are bad}] \leqslant 2c^{-2}$, we have $\Pr[\widehat{I} \text{ and } \widehat{U} \text{ are good}] \geqslant 1 - 2c^{-2}$, and so

$$\frac{\mathbb{E}[\widehat{I}] - c\,\sigma_{\mathrm{i}}}{\mathbb{E}[\widehat{U}] + c\,\sigma_{\mathrm{u}}}(1 - 2c^{-2}) \leqslant T_1 \leqslant \frac{\mathbb{E}[\widehat{I}] + c\,\sigma_{\mathrm{i}}}{\mathbb{E}[\widehat{U}] - c\,\sigma_{\mathrm{u}}}.$$

Now, observe that $\frac{a}{b} \geqslant \frac{a-x}{b-x}$, for $0 < a \leqslant b$ and $0 \leqslant x < b$ and $\mathbb{E}[\widehat{I}] - c\,\sigma_{\mathrm{i}} \leqslant \mathbb{E}[\widehat{U}] + c\,\sigma_{\mathrm{u}}$, since $\mathbb{E}[\widehat{I}] \leqslant \mathbb{E}[\widehat{U}]$ and $c \geqslant 0$.

$$\mathbb{E}[\widehat{R}] - \frac{\mathbb{E}[\widehat{I}]}{\mathbb{E}[\widehat{U}]} = T_1 + T_2 - \frac{\mathbb{E}[\widehat{I}]}{\mathbb{E}[\widehat{U}]} \geqslant T_1 - \frac{\mathbb{E}[\widehat{I}]}{\mathbb{E}[\widehat{U}]} \geqslant \frac{\mathbb{E}[\widehat{I}] - c\,\sigma_{\mathrm{i}}}{\mathbb{E}[\widehat{U}] + c\,\sigma_{\mathrm{u}}}\left(1 - 2c^{-2}\right) - \frac{\mathbb{E}[\widehat{I}]}{\mathbb{E}[\widehat{U}]} \geqslant \frac{\mathbb{E}[\widehat{I}] - c(\sigma_{\mathrm{i}} + \sigma_{\mathrm{u}})}{\mathbb{E}[\widehat{U}]}(1 - 2c^{-2}) - \frac{\mathbb{E}[\widehat{I}]}{\mathbb{E}[\widehat{U}]}. \quad (11)$$

Observe that for all $x > 0$ and $y > 0$, $\sqrt{x} + \sqrt{y} \leqslant \sqrt{x+y} + \sqrt{x+y} = \sqrt{2(x+y)}$. Then, using Lemma 4, we get:

$$\sigma_{\mathrm{i}} + \sigma_{\mathrm{u}} \leqslant \sqrt{2(Var(\widehat{I}) + Var(\widehat{U}))} \leqslant \sqrt{80w^2L}.$$

Furthermore, since every w consecutive k-mers have at least one minimizer, $\widehat{U} \geqslant L/w$, and so

$$\frac{c(\sigma_{\mathrm{i}} + \sigma_{\mathrm{u}})}{\mathbb{E}[\widehat{U}]} \leqslant \frac{L^{1/6}\sqrt{80w^2L}}{L/w} \leqslant \frac{\sqrt{80}w^2}{\sqrt[3]{L}}. \quad (12)$$

Plugging this bound into Equation (11) we get

$$\mathbb{E}[\widehat{R}] - \frac{\mathbb{E}[\widehat{I}]}{\mathbb{E}[\widehat{U}]} \geqslant \left(\frac{\mathbb{E}[\widehat{I}]}{\mathbb{E}[\widehat{U}]} - \frac{\sqrt{80}w^2}{\sqrt[3]{L}}\right)\left(1 - \frac{2}{\sqrt[3]{L}}\right) - \frac{\mathbb{E}[\widehat{I}]}{\mathbb{E}[\widehat{U}]} = -\frac{\sqrt{80}w^2}{\sqrt[3]{L}}\left(1 - \frac{2}{\sqrt[3]{L}}\right) - \frac{\mathbb{E}[\widehat{I}]}{\mathbb{E}[\widehat{U}]}\frac{2}{\sqrt[3]{L}} \geqslant -\frac{\sqrt{80}w^2}{\sqrt[3]{L}} - \frac{2}{\sqrt[3]{L}} \geqslant -\frac{11w^2}{\sqrt[3]{L}}. \quad (13)$$

To derive the upper bound for $\mathbb{E}[\widehat{R}] - \mathbb{E}[\widehat{I}]/\mathbb{E}[\widehat{U}]$, we first consider the case when $\mathbb{E}[\widehat{U}] - \mathbb{E}[\widehat{I}] < c\,(\sigma_{\mathrm{i}} + \sigma_{\mathrm{u}})$. Under this assumption,

$$\mathbb{E}[\widehat{R}] - \frac{\mathbb{E}[\widehat{I}]}{\mathbb{E}[\widehat{U}]} \leqslant 1 - \frac{\mathbb{E}[\widehat{I}]}{\mathbb{E}[\widehat{U}]} = \frac{\mathbb{E}[\widehat{U}] - \mathbb{E}[\widehat{I}]}{\mathbb{E}[\widehat{U}]} < \frac{c\,(\sigma_{\mathrm{i}} + \sigma_{\mathrm{u}})}{\mathbb{E}[\widehat{U}]} \leqslant \frac{\sqrt{80}w^2}{\sqrt[3]{L}},$$

where the last inequality follows from Equation (12)

Now consider the case when $\mathbb{E}[\widehat{U}] - \mathbb{E}[\widehat{I}] \geqslant c\,(\sigma_{\text{i}} + \sigma_{\text{u}})$. Using the fact that $\frac{a}{b} \leqslant \frac{a+x}{b+x}$, for $0 < a \leqslant b$ and $x \geqslant 0$, we obtain

$$T_1 \leqslant \frac{\mathbb{E}[\widehat{I}] + c\sigma_{\text{i}}}{\mathbb{E}[\widehat{U}] - c\,\sigma_{\text{u}}} \leqslant \frac{\mathbb{E}[\widehat{I}] + c(\sigma_{\text{i}} + \sigma_{\text{u}})}{\mathbb{E}[\widehat{U}]} \leqslant \frac{\mathbb{E}[\widehat{I}]}{\mathbb{E}[\widehat{U}]} + \frac{\sqrt{80}w^2}{\sqrt[3]{L}},$$

where the last inequality follows from Equation (12).

Putting the upper bounds on T_1 and T_2 together we get

$$\mathbb{E}[\widehat{R}] - \frac{\mathbb{E}[\widehat{I}]}{\mathbb{E}[\widehat{U}]} = T_1 + T_2 - \frac{\mathbb{E}[\widehat{I}]}{\mathbb{E}[\widehat{U}]} \leqslant \frac{\sqrt{80}w^2}{\sqrt[3]{L}} + 2c^{-2} \leqslant \frac{\sqrt{80}w^2 + 2}{\sqrt[3]{L}} \leqslant \frac{11w^2}{\sqrt[3]{L}}.$$

Combined with Equation (13) this implies the result. □

A.2.3 Proof of Theorem 1

To prove Theorem 1, we need to relate the bound on $\widehat{J}(A, B; w)$ given by Lemma 2 to the values of $J(A, B)$. We first express $J(A, B)$ in terms of configuration numbers. Let $\mathcal{D}(A, B; w) = \sum_{s=0}^{w} N(⟦*, 0; *, 0; s⟧)$. Note that, except near the start of the sequences, $A_i = B_j$ if and only if $(i - w, j - w)$ are in a configuration $⟦*, 0; *, 0; s⟧$. Therefore, $\mathcal{D}(A, B; w)$ is approximately $I(A, B)$. Formally, we can prove:

Lemma 6. *If A and B are padded, then $\mathcal{D}(A, B; w) = I(A, B)$ and $J(A, B) = \frac{\mathcal{D}(A,B;w)}{2L - \mathcal{D}(A,B;w)}$. More generally,*

(i) $\mathcal{D}(A, B; w) \leqslant I(A, B) \leqslant \mathcal{D}(A, B; w) + 2w$;
(ii) $\frac{\mathcal{D}(A,B;w)}{2L - \mathcal{D}(A,B;w)} \leqslant J(A, B) \leqslant \frac{\mathcal{D}(A,B;w)}{2L - \mathcal{D}(A,B;w)} + \frac{4w}{L}$.

Proof. Observe that for $i \in [w, L - 1]$ and $j \in [w, L - 1]$, we have $A_i = B_j$ if and only if $(i - w, j - w)$ are in a configuration with $C_{\text{a,right}} = C_{\text{b,right}} = 0$. In the case that A and B are padded, then $I = \mathcal{D}$ and $J = \frac{I}{2L - I} = \frac{\mathcal{D}}{2L - \mathcal{D}}$. In general, the number of (i, j) pairs for which $A_i = B_j$ and either $i \in [0, w - 1]$ or $j \in [0, w - 1]$ is at most $2w$. Hence, $\mathcal{D} \leqslant I \leqslant \mathcal{D} + 2w$. For the J lower bound, $J = \frac{I}{2L - I} \geqslant \frac{\mathcal{D}}{2L - \mathcal{D}}$. For the J upper bound, $J \leqslant \frac{\mathcal{D} + 2w}{2L - \mathcal{D} - 2w}$. When $\mathcal{D} + 2w \leqslant L$, then

$$J(A, B) \leqslant \frac{\mathcal{D} + 2w + 2w}{2L - \mathcal{D} - 2w + 2w} = \frac{\mathcal{D}}{2L - \mathcal{D}} + \frac{4w}{2L - \mathcal{D}} \leqslant \frac{\mathcal{D}}{2L - \mathcal{D}} + \frac{4w}{L}.$$

When $\mathcal{D} + 2w > L$, then

$$\frac{\mathcal{D}}{2L - \mathcal{D}} + \frac{4w}{L} \geqslant \frac{L - 2w}{L + 2w} + \frac{4w}{L} \geqslant \frac{L - 4w}{L} + \frac{4w}{L} = 1 \geqslant J.$$

□

We note that it is possible to derive exact expressions for $I(A, B; w)$ and $J(A, B; w)$ for the non-padded case as well; however, doing so is not necessary for our purposes and would just introduce (even more) burdensome notation. Next, we need to prove two facts:

Fact 2. $\mathcal{C}(A, B; w) \leqslant \frac{2L}{w+1}$.

Proof. By Lemma 1, the definition of $\widehat{I}$, and Fact 4, we have $\mathcal{C}(A, B; w) \leqslant \mathbb{E}[\widehat{I}(A, B; w)] = \sum_{p=0}^{L-1} \mathbb{E}[M_p^A] \leqslant \frac{2L}{w+1}$. □

Fact 3. *For all $y > 20$ and $0 < x \leqslant y/2$, $\frac{x+2}{y-x-10} - \frac{x}{y-x} \leqslant \frac{12}{y-y}$.*

Proof. Note that under the given assumptions, $y - x \geqslant y/2 > 0$ and $y - x - 10 \geqslant y/2 - 10 > 0$. Therefore,

$$\frac{x + 2}{y - x - 10} - \frac{x}{y - x} = \frac{2y + 8x}{(y - x)(y - x - 10)} \leqslant \frac{2y + \frac{8y}{2}}{\frac{y}{2}(\frac{y}{2} - 10)} = \frac{12}{y - 5}.$$

□

Now, we are ready to prove Theorem 1

Theorem 1. *Let $w \geqslant 2$, $k \geqslant 2$, and $L \geqslant 7(w + 1)$ be integers. Let A and B be two duplicate-free sequences, each consisting of L k-mers. Then there exists $\varepsilon \in [0, \frac{15w^2}{\sqrt[3]{L}}]$ such that*

$$\mathcal{B}(A, B, w) - \varepsilon \leqslant \mathbb{E}[\widehat{J}(A, B; w)] - J(A, B) \leqslant \mathcal{B}(A, B, w) + \varepsilon.$$

Proof. We prove the upper bound first. From Lemmas 2 and 6, we know that

$$\mathbb{E}[\widehat{J}(A, B; w)] - J(A, B) \leqslant \frac{\mathcal{C}(A, B; w)}{\frac{4L}{w+1} - \mathcal{C}(A, B; w)} + \frac{15w^2}{\sqrt[3]{L}} - \frac{\mathcal{D}(A, B; w)}{2L - \mathcal{D}(A, B; w)} = \mathcal{B}(A, B; w) + \frac{15w^2}{\sqrt[3]{L}}.$$

For the lower bound, we have

$$\begin{aligned}
\mathbb{E}[\widehat{J}(A,B;w)] - J(A,B) &= \frac{\mathcal{C}(A,B;w)}{\frac{4L}{w+1} - \mathcal{C}(A,B;w)} - \frac{11w^2}{\sqrt[3]{L}} - J(A,B) && \text{(Lemma 2)} \\
&\geqslant \frac{\mathcal{C}(A,B;w)}{\frac{4L}{w+1} - \mathcal{C}(A,B;w)} - \frac{11w^2}{\sqrt[3]{L}} - \frac{\mathcal{D}}{2L-\mathcal{D}} - \frac{4w}{L} && \text{(Lemma 6)} \\
&= \mathcal{B}(A,B;w) - \frac{11w^2}{\sqrt[3]{L}} - \frac{4w}{L} \\
&\geqslant \mathcal{B}(A,B;w) - \frac{11w^2+4w}{\sqrt[3]{L}} \geqslant \mathcal{B}(A,B;w) - \frac{11w^2+2w^2}{\sqrt[3]{L}} \geqslant \mathcal{B}(A,B;w) - \frac{13w^2}{\sqrt[3]{L}},
\end{aligned}$$

as claimed. □

A.3 Proof of Theorem 2

Theorem 2. *Let $w \geqslant 2$, $k \geqslant 2$, and $L \geqslant 7(w+1)$ be integers. Let A and B be two duplicate-free padded sequences, each consisting of L k-mers. Then $\mathcal{B}(A,B;w) < 0$ unless $J(A,B) = 0$; when $J(A,B) = 0$, we have $\mathcal{B}(A,B;w) = 0$.*

Proof. We omit the parameters A, B and w from the following for conciseness. Let $d = \frac{2}{w+1}$. Observe that the following statements are equivalent:

$$\begin{aligned}
\mathcal{B} \leqslant 0 &\Leftrightarrow \frac{\mathcal{C}}{2dL-\mathcal{C}} \leqslant \frac{\mathcal{D}}{2L-\mathcal{D}} \\
&\Leftrightarrow \mathcal{C}(2L-\mathcal{D}) \leqslant \mathcal{D}(2dL-\mathcal{C}) \\
&\Leftrightarrow 2L\mathcal{C} - \mathcal{D}\mathcal{C} \leqslant 2dL\mathcal{D} - \mathcal{D}\mathcal{C} \\
&\Leftrightarrow 2L\mathcal{C} \leqslant 2dL\mathcal{D} \\
&\Leftrightarrow \mathcal{C} \leqslant d\mathcal{D}
\end{aligned}$$

Note that for the second equivalence, we rely on the fact $\mathcal{B}(A,B;w)$ is well defined and its denominators are not zero. In other words, 1) $2L-\mathcal{D} > 0$ because $\mathcal{D} \leqslant L$ (by definition) and 2) $2dL - \mathcal{C} > 0$ because $\mathcal{C} \leqslant dL$ (by Fact 2).

We now need to show that $\mathcal{C} \leqslant d\mathcal{D}$. We have

$$\begin{aligned}
\mathcal{C} &\leqslant \mathbb{E}[\widehat{I}] && \text{(by Lemma 1)} \\
&= \sum_{p=0}^{L-1}\sum_{q=0}^{L-1} \mathbb{1}(A_p = B_q)\Pr[M_p^A = 1, M_q^B = 1] \\
&= \sum_{p=0}^{L-1}\sum_{q=0}^{L-1} \mathbb{1}(A_p = B_q)\Pr[M_p^A = 1 \mid M_q^B = 1]\Pr[M_q^B = 1] \\
&= \sum_{p=0}^{L-1}\sum_{q=0}^{L-1} \mathbb{1}(A_p = B_q)\Pr[M_p^A = 1 \mid M_q^B = 1]d && (14) \\
&\leqslant Id \\
&= d\mathcal{D} && \text{(by Lemma 6)}
\end{aligned}$$

Note that Equation (14) follows because of the fact that A and B are padded and Fact 4. Next, observe that since all the terms in Equation (14) are positive, the only way to have equality with Id is if each term $\Pr[M_p^A = 1 \mid M_q^B = 1]$ is 1. We claim this can only happen if there are no shared k-mers between A and B, i.e. when $J(A,B) = 0$. Otherwise, take the leftmost shared k-mer in A. The window to its left in A will be assigned hash values that are independent of the hash values in B; therefore, $\Pr[M_p^A = 1 \mid M_q^B = 1]$ cannot be 1. Thus, if A and B share at least one k-mer, we get the stronger statement that $\mathbb{E}[\widehat{I}(A,B;w)] < Id$. This in turn implies that $\mathcal{C} < d\mathcal{D}$, which propagates to imply that $\mathcal{B} < 0$. □

A.4 Proof of Theorem 3

Theorem 3. *Let $w \geqslant 2$, $k \geqslant 2$, and $L \geqslant 7(w+1)$ be integers. Let A and B be two duplicate-free, padded, sparsely-matched sequences, each consisting of L k-mers. Then $\mathcal{B}(A,B;w) \leqslant -J(A,B)\frac{3w^2-3w}{8w^2-2}$.*

Proof. This proof simply counts the configuration numbers and then applies definitions and Theorem 1. We will first count the configuration numbers. Let us call $[\![2,2;2,2;0]\!]$ the *empty* configuration. Note that the terms involving the number of empty configurations cancel out in the equation for $\mathcal{C}$ and hence we do not need to count them. Observe, by the condition of the theorem, that a configuration (i,j) that is non-empty

must contain exactly one pair $p \in [i, i+w]$ and $q \in [j, j+w]$ such that $A_p = B_q$. Therefore, to count the number of non-empty configurations, it suffices to count, for every $p \in [0, L-1]$ and $q \in [0, L-1]$ such that $A_p = B_q$, the types of configurations (i, j) for $i \in [p-w, p]$ and $j \in [q-w, q]$. Following a case analysis, we get one configuration of $[\![2,0;2,0;1]\!]$, $w-1$ configurations of $[\![2,1;2,2;1]\!]$, $w-1$ configurations of $[\![2,2;2,1;1]\!]$, $(w-1)^2$ configurations of $[\![2,2;2,2;1]\!]$, one configuration of $[\![0,2;0,2;0]\!]$, w configurations of $[\![1,2;2,2;0]\!]$, and w configurations of $[\![2,2;1,2;0]\!]$. Recall that $I = I(A,B)$ is the number of shared k-mers between A and B. Summing over all I values of p, we then get the non-zero configuration number of non-empty configurations are

$$
\begin{aligned}
N([\![2,0;2,0;1]\!]) &= I \\
N([\![2,1;2,2;1]\!]) &= I(w-1) \\
N([\![2,2;2,1;1]\!]) &= I(w-1) \\
N([\![2,2;2,2;1]\!]) &= I(w-1)^2 \\
N([\![1,2;2,2;0]\!]) &= Iw \\
N([\![2,2;1,2;0]\!]) &= Iw \\
N([\![0,2;0,2;0]\!]) &= I.
\end{aligned}
$$

We then plug these into the definition of $\mathcal{C}$ to get that $\mathcal{C}(A,B;w) = \beta I$, where $\beta = \frac{5w-2}{4w^2-1}$. By Lemma 6, $\mathcal{D}(A,B;w) = I$. Let $d \triangleq 2/(w+1)$. Note that $\beta - d = \frac{3w^2-3w}{-(w+1)(4w^2-1)} \leqslant 0$. Using these facts, we can now derive

$$
\begin{aligned}
\mathcal{B}(A,B;w) &\triangleq \frac{(w+1)\mathcal{C}(A,B;w)}{4L-(w+1)\mathcal{C}(A,B;w)} - \frac{\mathcal{D}(A,B;w)}{2L-\mathcal{D}(A,B;w)} = \frac{\mathcal{C}(A,B;w)}{2dL-\mathcal{C}(A,B;w)} - \frac{I}{2L-I} = \frac{\beta I}{2dL-\beta I} - \frac{I}{2L-I} \\
&= \frac{(2L-I)\beta I - 2dLI + \beta I^2}{(2dL-\beta I)(2L-I)} = \frac{2L\beta I - 2dLI}{(2dL-\beta I)(2L-I)} = J(A,B)\frac{2L\beta - 2dL}{2dL-\beta I} = J(A,B)\frac{2L(\beta-d)}{2dL-\beta I}.
\end{aligned}
$$

Note that because $\beta - d \leqslant 0$, $\mathcal{B}(A,B;w) \leqslant 0$. Then, using the fact that $\beta > 0$ and $I > 0$, we get

$$
\mathcal{B}(A,B;w) \leqslant J(A,B)\frac{2L(\beta-d)}{2dL} = J(A,B)\frac{3w^2-3w}{-2(4w^2-1)}.
$$

□

A.5 Proof of Theorem 4

Theorem 4. *Let $2 \leqslant w < k$, $g > w + 2k$, and $L = \ell g + k$ for some integer $\ell \geqslant 1$. Let A and B be two duplicate-free sequences with L k-mers such that A and B are identical except that the nucleotides at positions $k-1+ig$, for $i = 0, \ldots, \ell$, are mutated. Then,*

$$
\mathcal{B}(A,B;w) = \frac{2\ell(\ell g+k)h(w)}{(\ell(g+k)+2k-\ell h(w))(\ell(g+k)+2k)},
$$

where $h(w) = \frac{(w+1)(1-2(H_{2w}-H_w))}{2}$ and $H_n = \sum_{j=1}^{n}\frac{1}{j}$ denotes the n-th Harmonic number.

Proof. Let

$$
\begin{aligned}
W(s) = {} & t_0(N([\![1,0;1,0;s]\!]) + N([\![1,0;2,0;s]\!]) + N([\![2,0;1,0;s]\!])) \\
&+ t_1(N([\![2,\{1,2\};1,1;s]\!]) + N([\![1,1;2,\{1,2\};s]\!]) + 2wN([\![0,0;0,0;s]\!])) \\
&+ t_1 s(N([\![0,1;0,1;s]\!]) + N([\![0,1;0,2;s]\!]) + N([\![0,2;0,1;s]\!]) + N([\![0,2;0,2;s]\!])) \\
&+ t_2(2sN([\![2,2;2,2;s]\!]) + 4wN([\![2,1;2,1;s]\!]) + (6w-s+(2w-s)^2)N([\![2,0;2,0;s]\!])) \\
&+ t_2(s+2w)(N([\![2,1;2,2;s]\!]) + N([\![2,2;2,1;s]\!]))
\end{aligned}
$$

so that $\mathcal{C}(A,B;w) = \sum_{s=0}^{w} W(s)$. In our setting, the configuration counts are such that the following holds:

Fact 6.

$$
W(s) = \begin{cases} 0 & \textit{if } s = 0; \\ \frac{2\ell(g-w-k)}{w+1} + \frac{\ell(w+5)}{(w+1)(w+2)} & \textit{if } s = w; \\ \ell s t_1 + \ell t_2(6w+8w^2-s(s+6w+1)) & \textit{if } 1 \leqslant s \leqslant w-1. \end{cases}
$$

From this fact, which we prove later, we get that $\mathcal{C}(A,B;w) = d\ell(g-k) + \ell f(w)$, where $d = 2/(w+1)$ and

$$
f(w) = -\frac{2w}{w+1} + \frac{w+5}{(w+1)(w+2)} + \sum_{s=1}^{w-1} st_1 + t_2(6w+8w^2-s(s+6w+1)).
$$

configuration	count	reason for 0	configuration	count	reason for 0
$[\![0,2;0,2;<w]\!]$	ℓ	N/A	$[\![0,2;0,2;w]\!]$	0	TOO-FULL
$[\![2,2;2,2;>0]\!]$	0	see text	$[\![2,1;2,1;s]\!]$	0	CROSS
$[\![0,0;0,0;<w]\!]$	0	see text	$[\![0,0;0,0;w]\!]$	$\ell(g-w-k)$	N/A
$[\![1,0;1,0;s]\!]$	0	VERT	$[\![2,0;1,0;s]\!]$	0	VERT
$[\![1,0;2,0;s]\!]$	0	VERT	$[\![2,0;2,0;0]\!]$	0	TOO-EMPTY
$[\![2,0;2,0;>0]\!]$	ℓ	N/A	$[\![0,1;0,1;s]\!]$	0	VERT
$[\![0,2;0,1;s]\!]$	0	VERT	$[\![2,1;1,1;s]\!]$	0	CROSS
$[\![2,2;1,1;s]\!]$	0	CROSS	$[\![2,1;2,1;s]\!]$	0	CROSS
$[\![2,2;2,1;0]\!]$	0	TOO-EMPTY	$[\![2,2;2,1;1\cdots w-1]\!]$	$\ell(w-s)$	N/A
$[\![2,2;2,1;w]\!]$	0	TOO-FULL	$[\![0,1;0,2;s]\!]$	0	VERT
$[\![1,1;2,1;s]\!]$	0	CROSS	$[\![1,1;2,2;s]\!]$	0	CROSS
$[\![2,1;2,2;0]\!]$	0	TOO-EMPTY	$[\![2,1;2,2;1\cdots w-1]\!]$	$\ell(w-s)$	N/A
$[\![2,1;2,2;w]\!]$	0	TOO-FULL			

Table S3. Non-empty configurations appearing in the definition of $\mathcal{C}$, along with their counts in the context of Theorem 4 as well as why the counts are zero, if applicable. The reasons are explained in the proof of Fact 8.

Note that since there are no matches in the first or the last k-mers and $k \geqslant w$, we have by Lemma 6 that $I = |A \cap B| = \mathcal{D}(A,B;w) = \ell(g-k)$ and so

$$\mathcal{C}(A,B;w) = dI + \ell f(w),$$

From the definition of $\mathcal{B}(A,B;w)$, we then have

$$\mathcal{B}(A,B;w) = \frac{\mathcal{C}}{2dL-\mathcal{C}} - \frac{I}{2L-I} = \frac{I + \frac{\ell f(w)}{d}}{2L - I - \frac{\ell f(w)}{d}} - \frac{I}{2L-I} = \frac{2L\frac{\ell f(w)}{d}}{(2L-I-\frac{\ell f(w)}{d})(2L-I)}.$$

We also have the following closed form for $f(w)$ (which we prove later).

Fact 7. *For $n \geqslant 1$, let $H_n = \sum_{k=1}^{n} \frac{1}{k}$. Then, $f(w) = 1 - 2(H_{2w} - H_w)$.*

From this, combined with the facts that $L = \ell g + k$ and $I = \ell(g-k)$, and letting $h(w) = \frac{(w+1)(1-2(H_{2w}-H_w))}{2}$, we get

$$\mathcal{B}(A,B;w) = \frac{2\ell(\ell g+k)h(w)}{(\ell(g+k)+2k-\ell h(w))(\ell(g+k)+2k)},$$

as claimed. □

It remains for use to provide the proofs of Facts 6 and 7. Fact 6 is a direct consequence of the following configuration counts.

Fact 8. *In the setting of Theorem 4, we have*

(i)$N([\![0,0;0,0;w]\!]) = l(g-w-k)$*;*
(ii)$N([\![0,2;0,2;\{0,\cdots,w-1\}]\!]) = l$*;*
(iii)$N([\![2,0;2,0;\{1,\cdots,w\}]\!]) = l$*;*
(iv)$N([\![2,1;2,2;\{1,\cdots,w-1\}]\!]) = l(w-s)$*;*
(v)$N([\![2,2;2,1;\{1,\cdots,w-1\}]\!]) = l(w-s)$*.*

For any other configuration c that could contribute to $\mathcal{C}(A,B;w)$, we have $N(c) = 0$ or $c = [\![2,2;2,2;0]\!]$.

Proof. We will refer to $[\![2,2;2,2;0]\!]$ as the *empty* configuration. Table S3 lists all non-empty configurations that appear in the definition of $\mathcal{C}$. Sometimes, a configuration type is further sub-divided according to different values of s. We will show that the counts in the table are correct, which will prove the Theorem.

The rows that whose reason is VERT have configurations that match $[\![*,*;1,0;s]\!]$, $[\![*,*;0,1;s]\!]$, $[\![1,0;*,*;s]\!]$, or $[\![0,1;*,*;s]\!]$. These configurations never occur because in our setting, all the matches are parallel to each other (i.e. if $A_i = B_j$ and $A_{i'} = B_{j'}$, then $j - i = j' - i'$), while these configurations contain a 0 in one place (indicating that the matches are vertical, i.e. $A_i = B_j$ implies $i = j$) and a 1 in another (indicated that the matching edges are angled, i.e. $A_i = B_j$ implies $i \neq j$). The rows whose reason is CROSS have a configuration that matches $[\![1,*;1,*;s]\!]$, $[\![*,1;*,1;s]\!]$, $[\![1,1;*,*;s]\!]$, or $[\![*,*;1,1;s]\!]$. These configurations never occur because the 1s indicate conflicting angles for the matches — they should either slant left (e.g. $i > j$) or right (e.g. $i < j$), but cannot do both. Note that for rows that could be categorized as both VERT and CROSS, the reason in the Table is arbitrarily chosen from those two. The rows whose reason is TOO-FULL have a configuration that matches $[\![*,2;*,*;w]\!]$ or $[\![*,*;*,2;w]\!]$. These configurations can never occur because the presence of the 2 indicates that either A_{i+w} or B_{j+w} is not involved in a match, making it impossible that $S(i+1,j+1,w) = w$. The rows whose reason is TOO-EMPTY have a configuration that matches $[\![*,*;*,\{0,1\};0]\!]$ or $[\![*,\{0,1\};*,*;0]\!]$. These configurations can never occur because the presence of the 0 or 1 indicates that either A_{i+w} or B_{j+w} is involved in a match, making it impossible that $S(i+1,j+1,w) = 0$.

By the definition of A and B from Theorem 4, we have alternating runs of k mismatches followed by $g - k$ matches, with k mismatches at the end. Therefore, we have $\ell + 1$ blocks of k mismatches, at $i \in \{ig, ..., ig + k - 1 | 0 \leqslant i \leqslant \ell\}$, and we have ℓ blocks of $g - k$ matches, at $i \in \{ig + k, ..., (i + 1)g - 1 | 0 \leqslant i < \ell\}$. We will refer to the latter as *match-blocks*.

Recall that configuration windows are of length $w + 1$. Because $k > w$, no window can contain matches from more than one match-block. Moreover, any configurations involving an i or j in the first match-block will occur again in each other match-block, at the same coordinates modulo g. Thus it is enough to consider only the first match-block, and multiply the resulting counts by ℓ. We therefore restrict ourselves to the first match-block in the following discussion, and note that the leftmost match is at position k and the rightmost match is at $g - 1$.

Let us consider the configurations that are $[\![2, 2; 2, 2; > 0]\!]$. In this case, $A_i \neq B_j$ and $A_{i+w} \neq B_{j+w}$, and there is some $i' \in [i+1, i+w-1]$ and $j' \in [j + 1, j + w - 1]$ such that $A_{i'} = B_{j'}$. This match must be part of match block, and in our setting, a match block has width $g - k$. This is more than w, making it impossible that $A_i \neq B_j$ and $A_{i+w} \neq B_{j+w}$. Hence $N([\![2, 2; 2, 2; > 0]\!]) = 0$.

Let us consider the configurations that are $[\![0, 0; 0, 0; s]\!]$. In these configuration, $i = j$, $A_i = B_j$, and $A_{i+w} = B_{j+w}$. A configuration window of width $w + 1$ cannot span more than one match block, since $g > w$. Therefore, $A_{i+\delta} = B_{j+\delta}$ for all $0 \leqslant \delta \leqslant w$. Hence, the number of configurations with $s < w$ is 0. For $s = w$, Figure S2A shows all the configurations that are $[\![0, 0; 0, 0; w]\!]$. We have that $i \in [k, g - w - 1]$, resulting in $g - w - k$ possible windows with this configuration, in one match block

Let us consider the configurations that are $[\![0, 2; 0, 2; s]\!]$ for $0 \leqslant s \leqslant w - 1$. In this situation, $A_i = B_j$ and hence $i = j$. The match block containing this match ends before A_{i+w}, since $A_{i+w} \neq B_{j+w}$ in this configuration. Then the rightmost match, $A_{g-1} = B_{g-1}$, must be somewhere in the window, other than at $i + w$. To get s matches, $g - 1 = i + s$ and thus $i = g - s - 1$. Therefore, $N([\![0, 2; 0, 2; s]\!]) = 1$ for each $s \in [0, w - 1]$. Figure S2B shows how this configuration looks like. The top and bottom drawings show the two end cases, while the middle drawing demonstrates the general case.

Let us consider the configurations that are $[\![2, 0; 2, 0; s]\!]$ for $1 \leqslant s \leqslant w$. The case is mostly symmetric to the previous one. In this situation, $A_{i+w} = B_{j+w}$ and hence $i = j$. The match block containing this match begins after A_i, since $A_i \neq B_j$ in this configuration. The leftmost match in the match-block, A_k, must be somewhere in the window other than at A_i. To get s matches, $k = (i + w) - (s - 1)$ and thus $i = k - w + s - 1$. Therefore $N([\![2, 0; 2, 0; s]\!]) = 1$ for each $s \in [1, w]$. Figure S2C shows how this configurations looks like. The top and bottom drawings show the two end cases, while the middle drawing demonstrates the general case.

Let us consider the configurations that are $[\![2, 1; 2, 2; s]\!]$ for $1 \leqslant s \leqslant w - 1$. Figure S2D shows all the configurations. There are several possibilities for each s. For $s = 3$, the top and bottom drawings show the two end cases, while the middle drawing demonstrates the general case. Because $C_{\text{a,right}} = 1$, $A_{i+w} \in \{B_{j+1}, \ldots, B_{j+w-1}\}$ and $j > i$. Since $C_{\text{a,left}} = C_{\text{b,left}} = 2$, $A_i \neq B_j$, and the leftmost match in the match-block, A_k, must be somewhere in the window, other than at i. To get s matches, $k = (i + w) - (s - 1)$ and thus $i = k - w + s - 1$. The window for B can be positioned so that the leftmost match occurs in $\{j + 1, \ldots, j + w - s\}$. Since this corresponds to A_k, we have $k \in \{j + 1, \ldots, j + w - s\}$, which can be restated as $(i + w) - (s - 1) \in \{j + 1, \ldots, j + w - s\}$. We can in turn restate this as $i \in \{j - w + s, \ldots, j - 1)\}$ and thus $j \in \{i + 1, \ldots, i + w - s)\}$. Therefore, $N([\![2, 1; 2, 2; s]\!]) = w - s$ for each $s \in [1, w - 1]$.

Finally, we consider the configurations that are $[\![2, 2; 1, 2; s]\!]$ for $1 \leqslant s \leqslant w - 1$. This case is symmetrical to the above case, by swapping the roles of A and B in the definition of the configurations. Therefore, $N([\![2, 2; 1, 2; s]\!]) = w - s$ for each $1 \leqslant s \leqslant w - 1$. □

We are now ready to prove Fact 6.

Fact 6.

$$W(s) = \begin{cases} 0 & \textit{if } s = 0; \\ \frac{2\ell(g-w-k)}{w+1} + \frac{\ell(w+5)}{(w+1)(w+2)} & \textit{if } s = w; \\ \ell s t_1 + \ell t_2(6w + 8w^2 - s(s + 6w + 1)) & \textit{if } 1 \leqslant s \leqslant w - 1. \end{cases}$$

Proof. Let us consider first the $s = 0$ case. By Fact 8, the only two configurations with $s = 0$ and with non zero counts are $[\![2, 2; 2, 2; 0]\!]$ and $[\![0, 2; 0, 2; 0]\!]$. However, both of those terms are multiplied by s in $W(0)$, hence we have $W(0) = 0$.

Let us consider next the $s = w$ case. For this value of s, by Fact 8, we have $N([\![0, 0; 0, 0; w]\!]) = l(g - w - k)$ and $N([\![2, 0; 2, 0; w]\!]) = l$; all other configurations that may contribute to $\mathcal{C}(A, B; w)$ have zero counts.

At $s = w$, $[\![0, 0; 0, 0; w]\!]$ has coefficient $\frac{2}{w+1}$ and $[\![2, 0; 2, 0; w]\!]$ has coefficient $\frac{w+5}{(w+1)(w+2)}$. Hence

$$W(w) = \frac{2l(g - w - k)}{w + 1} + \frac{l(w + 5)}{(w + 1)(w + 2)}.$$

Finally, when $1 \leqslant s \leqslant w - 1$, again by Fact 8, we have

$$N([\![0, 2; 0, 2; s]\!]) = N([\![2, 0; 2, 0; s]\!]) = l,$$

$$N([\![2, 1; 2, 2; s]\!]) = N([\![2, 2; 2, 1; s]\!]) = l(w - s),$$

and all other configurations do not contribute to W. Now, the coefficient of $N([\![0, 2; 0, 2; s]\!])$ in W is st_1, the coefficient of $N([\![2, 0; 2, 0; s]\!])$ in W is $t_2(6w - s + (2w - s)^2)$, and the coefficient of $N([\![2, 1; 2, 2; s]\!])$ and $N([\![2, 2; 2, 1; s]\!])$ in W is $t_2(s + 2w)$. Combining this, we obtain

$$W(s) = \ell s t_1 + \ell t_2(6w - s + (2w - s)^2) + 2\ell(w - s)(s + 2w)t_2 = \ell s t_1 + \ell t_2(6w + 8w^2 - s(s + 6w + 1))$$

as claimed. □

We conclude this section with the proof of Fact 7.

Fact 7. *For $n \geqslant 1$, let $H_n = \sum_{k=1}^{n} \frac{1}{k}$. Then, $f(w) = 1 - 2(H_{2w} - H_w)$.*

Proof. Recall that $f(w) \triangleq -\frac{2w}{w+1} + \frac{(w+5)}{(w+1)(w+2)} + \sum_{s=1}^{w-1} st_1 + t_2(6w + 8w^2 - s(s + 6w + 1))$. Let us rewrite $f(w)$ as

$$
\begin{aligned}
f(w) &= \frac{-2w(w+2) + w + 5}{(w+1)(w+2)} + \sum\nolimits_{s=1}^{w-1} s(2w - s + 2)t_2 + t_2(6w + 8w^2 - s(s + 6w + 1)) \\
&= \frac{-2w^2 - 3w + 5}{(w+1)(w+2)} + \sum\nolimits_{s=1}^{w-1} t_2(-s^2 + s(2w+2) + 6w + 8w^2 - s(s + 6w + 1)) \\
&= \frac{-2w^2 - 3w + 5}{(w+1)(w+2)} + \sum\nolimits_{s=1}^{w-1} t_2(-2s^2 + s(-4w + 1) + 6w + 8w^2) \\
&= \frac{-2w^2 - 3w + 5}{(w+1)(w+2)} - 2S_4 + (-4w + 1)S_2 + (6w + 8w^2)S_1,
\end{aligned}
$$

where $S_4 = \sum_{s=1}^{w-1} t_2 s^2$, $S_2 = \sum_{s=1}^{w-1} t_2 s$, and $S_1 = \sum_{s=1}^{w-1} t_2$. Let

$$T = -2S_4 + (-4w + 1)S_2 + (6w + 8w^2)S_1.$$

We will now reduce each of the sums.

$$S_1 = \sum_{s=1}^{w-1} t_2 = \sum_{s=1}^{w-1} \frac{1}{(2w-s)(2w-s+1)(2w-s+2)} = \sum_{i=w+1}^{2w-1} \frac{1}{i(i+1)(i+2)} = \sum_{i=1}^{2w-1} \frac{1}{i(i+1)(i+2)} - \sum_{i=1}^{w} \frac{1}{i(i+1)(i+2)}.$$

We now use the fact that $\sum_{k=1}^{n} \frac{1}{k(k+1)(k+2)} = \frac{n(n+3)}{4(n+1)(n+2)}$, which can be derived via partial fraction decomposition or induction. Then,

$$S_1 = \frac{(2w-1)(2w+2)}{4(2w)(2w+1)} - \frac{w(w+3)}{4(w+1)(w+2)} = \frac{(2w-1)(w+1)}{4w(2w+1)} - \frac{w(w+3)}{4(w+1)(w+2)}.$$

Proceeding similarly for the next component, we have:

$$S_2 = \sum_{s=1}^{w-1} \frac{s}{(2w-s)(2w-s+1)(2w-s+2)} = \sum_{i=w+1}^{2w-1} \frac{2w-i}{i(i+1)(i+2)} = 2wS_1 - \sum_{i=w+1}^{2w-1} \frac{1}{(i+1)(i+2)}.$$

Recalling that $\sum_{k=1}^{n} \frac{1}{(k+1)(k+2)} = \frac{n}{2(n+2)}$, we get

$$S_3 = \sum_{i=w+1}^{2w-1} \frac{1}{(i+1)(i+2)} = \sum_{i=1}^{2w-1} \frac{1}{(i+1)(i+2)} - \sum_{i=1}^{w} \frac{1}{(i+1)(i+2)} = \frac{2w-1}{2(2w+1)} - \frac{w}{2(w+2)}.$$

Hence

$$S_2 = 2wS_1 - S_3 = 2wS_1 - \frac{2w-1}{2(2w+1)} + \frac{w}{2(w+2)}.$$

Finally,

$$
\begin{aligned}
S_4 &= \sum_{s=1}^{w-1} \frac{s^2}{(2w-s)(2w-s+1)(2w-s+2)} = \sum_{i=w+1}^{2w-1} \frac{(2w-i)^2}{i(i+1)(i+2)} \\
&= 4w^2 \sum_{i=w+1}^{2w-1} \frac{1}{i(i+1)(i+2)} - 4w \sum_{i=w+1}^{2w-1} \frac{1}{(i+1)(i+2)} + \sum_{i=w+1}^{2w-1} \frac{i}{(i+1)(i+2)} \\
&= 4w^2 S_1 - 4wS_3 + \sum_{i=w+1}^{2w-1} \frac{i}{(i+1)(i+2)}.
\end{aligned}
$$

Using that $\sum_{k=1}^{n} \frac{k}{(k+1)(k+2)} = H_{n+1} + \frac{2}{n+2} - 2$ again via partial fraction decomposition or induction, we get

$$S_5 = \sum_{i=w+1}^{2w-1} \frac{i}{(i+1)(i+2)} = \sum_{i=1}^{2w-1} \frac{i}{(i+1)(i+2)} - \sum_{i=1}^{w} \frac{i}{(i+1)(i+2)} = H_{2w} - H_{w+1} + \frac{2}{2w+1} - \frac{2}{w+2}$$

$$= H_{2w} - H_w - \frac{1}{w+1} + \frac{2}{2w+1} - \frac{2}{w+2} = H_{2w} - H_w - \frac{3w+4}{(w+1)(w+2)} + \frac{2}{2w+1}.$$

Thus,

$$S_4 = 4w^2 S_1 - 4wS_3 + S_5.$$

Combining all of this, we get

$$\begin{aligned} T &= -2S_4 + (-4w+1)S_2 + (6w+8w^2)S_1 \\ &= -2(4w^2S_1 - 4wS_3 + S_5) + (-4w+1)(2wS_1 - S_3) + (6w+8w^2)S_1 \\ &= S_1(-8w^2+8w) + S_3(12w-1) - 2S_5. \end{aligned}$$

By using partial fraction decomposition, we can algebraically simplify each of the terms as follows:

$$\begin{aligned} S_1(-8w^2+8w) &= -8w(w-1)\left(\frac{(2w-1)(w+1)}{4w(2w+1)} - \frac{w(w+3)}{4(w+1)(w+2)}\right) = \frac{24}{w+2} - \frac{3}{2w+1} - \frac{8}{w+1} - 3, \\ S_3(12w-1) &= (12w-1)\left(\frac{2w-1}{2(2w+1)} - \frac{w}{2(w+2)}\right) = \frac{7}{2w+1} - \frac{25}{w+2} + 6, -2S_5 \\ &= -2\left(H_{2w} - H_w - \frac{3w+4}{(w+1)(w+2)} + \frac{2}{2w+1}\right) = \frac{4}{w+2} - \frac{4}{2w+1} + \frac{2}{w+1} - 2\left(H_{2w} - H_w\right). \end{aligned}$$

By plugging these expressions back into T, we get

$$T = \frac{3}{w+2} - \frac{6}{w+1} + 3 - 2\left(H_{2w} - H_w\right) = \frac{3(w^2+2w-1)}{(w+1)(w+2)} - 2\left(H_{2w} - H_w\right).$$

Now, we plug the value of T into $f(w)$ and it finishes the proof,

$$f(w) = \frac{-2w^2-3w+5}{(w+1)(w+2)} + T = \frac{-2w^2-3w+5}{(w+1)(w+2)} + \frac{3(w^2+2w-1)}{(w+1)(w+2)} - 2\left(H_{2w} - H_w\right) = 1 - 2\left(H_{2w} - H_w\right).$$

□

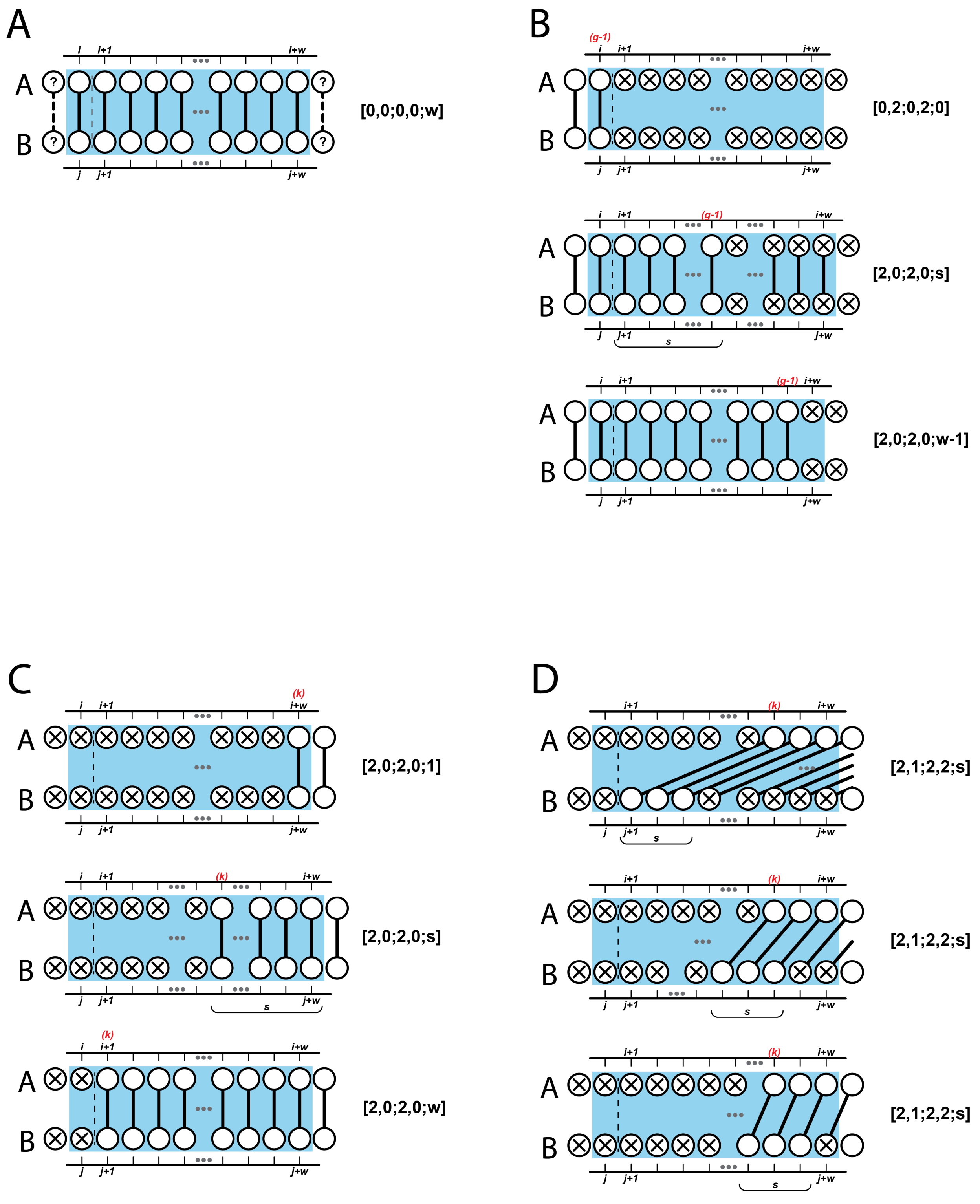

Fig. S2: Some of the configurations with non-zero counts in Fact 8.

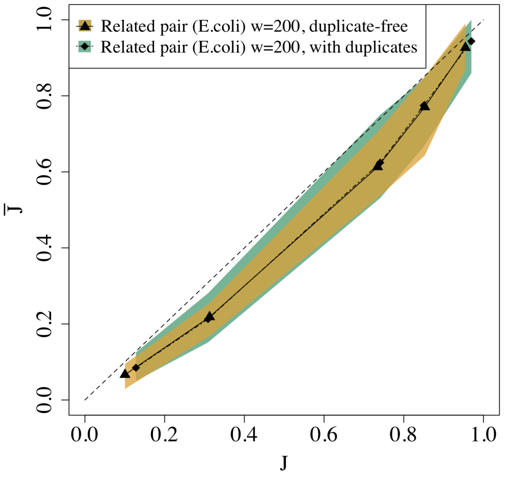

Fig. S3: Empirical bias for related sequence pairs, with and without duplicates. We set $k = 16$, $w = 200$, $L = 10000$, and $r_1 \in \{.001, .005, .01, .05, .1\}$, with one mutation replicate. The duplicate-free sequence is the same as in Figure 4. The sequence with duplicates was found by choosing 100 random L-k-mer sequences from E.coli and choosing from those the one with the most duplicate k-mers (it had 1,377 duplicates, or about 14%). The colored bands show the 2.5th and the 97.5th percentiles. The evenly dashed line shows the expected behavior of an unbiased estimator, with $\bar{J} = J$.

A.6 Experimental details

In this section, we provide some experimental details to aid reproducibility. The scripts to reproduce our experiments are available on our GitHub paper repository.

Generative models: When we generate an unrelated pair, we greedily extend each string from left to right. At each position, we choose, uniformly at random, one of the nucleotides that would not result in a k-mer we have already seen. If we get to a point where all the possible nucleotide extensions to a string are already present, we discard the string and start from the beginning. Though this sampling scheme is not guaranteed to terminate, we found that it always did in our experiments. We also verified that the Jaccard of the generated pair was close to the j that was used as a target. Under the assumptions that A and B are uniformly chosen, j should be the expected value under the generative process. Though it is not clear that the uniformity assumption holds in our generative process, we found that the true Jaccard was indeed very close to j in practice. In the related pair model, we also faced a possibility that after choosing to mutate a position, all the possible nucleotide substitutions would create a duplicate k-mer. In such a case, the position was left unchanged.

Mashmap divergence experiment: We sampled 100 substrings from the *E.coli* reference *E.Coli* download link, each of length $L = 10,000$ and, for each substring and for each $r_1 \in \{0.90, 0.95, 0.99\}$, generated a "read" which was the substring with $r_1 L$ positions randomly picked and mutated. We then mapped it with mashmap, and discarded any read for which mashmap did not correctly identify a unique and correct mapping location. Mashmap was run with default parameters of $k = 16$ and $w = 200$.

Correction formula to remove Poisson-approximation from Mash distance Let j be the observed Jaccard. Let A and B be two sequences generated using a simple mutation process, i.e. a substitution is created at every nucleotide with a given probability r_1 Blanca et al. [2021]. The method of moments Wasserman [2013] estimator for the sequence identity is $\widehat{\imath}_{\text{mom}} = (1-n/L)^{1/k}$, where n is the observed number of mutated k-mers Blanca et al. [2021]. In the simple mutation model, the observed Jaccard j is related to n via $j = \frac{L-n}{L+n}$, or, equivalently, $n = \frac{L(1-j)}{1+j}$ Blanca et al. [2021]. Putting this together, we get that $\widehat{\imath}_{\text{mom}} = (1 - \frac{1-j}{1+j})^{1/k} = \frac{2j}{1+j}^{1/k}$. On the other hand, the Mash distance estimator is $-\frac{1}{k}\log(\frac{2j}{1+j})$ (Formula 1 in Jain et al. [2017]), which equivalently translates to the identity estimator $\widehat{\imath}_{\text{mash}} = 1 + \frac{1}{k}\log(\frac{2j}{1+j})$. Combining the two, we get that $\widehat{\imath}_{\text{mash}} = 1 + \frac{1}{k}\log((\widehat{\imath}_{\text{mom}})^k)$. Solving for $\widehat{\imath}_{\text{mom}}$, we get the final correction formula: $\widehat{\imath}_{\text{mom}} = e^{\widehat{\imath}_{\text{mash}}-1}$.

Sliding read experiment: When choosing A, we avoided segments with any Ns or any duplicate k-mers. Any k-mers in B containing an N were hashed to the maximum hash value so as to avoid them being a minimizer. Also note that minimizers were computed separately for each B; thus, it is possible that the same k-mer might be a minimizer in one B but not a minimizer in a nearby B.

Empirical bias for related sequence pairs, allowing duplicates: The sequence chosen as the basis for the related experiment in Figure 4 did not contain duplicates, by chance. We wanted to check the extent to which this experiment would have been affected by duplicates. We chose 100 random sequences from E.coli and, from those, chose the one with the most duplicate k-mers. It had 1,377 duplicates, or about 14%. Figure S3 compares the bias for this sequence to the duplicate-free one in Figure 4. There is almost no visually discernible difference between the two.
